# A unique deubiquitinase that deconjugates phosphoribosyl-linked protein ubiquitination

**DOI:** 10.1038/cr.2017.66

**Published:** 2017-05-12

**Authors:** Jiazhang Qiu, Kaiwen Yu, Xiaowen Fei, Yao Liu, Ernesto S Nakayasu, Paul D Piehowski, Jared B Shaw, Kedar Puvar, Chittaranjan Das, Xiaoyun Liu, Zhao-Qing Luo

**Affiliations:** 1 Center of Infection and Immunity, The First Hospital, Jilin University, Changchun, Jilin 130001, China; 2 Purdue Institute for Inflammation, Immunology and Infectious Disease and Department of Biological Sciences, Purdue University, West Lafayette, IN 47907, USA; 3 Institute of Analytical Chemistry and Synthetic and Functional Biomolecules Center, College of Chemistry and Molecular Engineering, Peking University, Beijing 100871, China; 4 Department of Biochemistry and Molecular Biology, Hainan Medical College, Haikou, Hainan 571101, China; 5 Biological Sciences Division, Pacific Northwest National Laboratory, Richland, WA 99352, USA; 6 Environmental Molecular Sciences Laboratory, Pacific Northwest National Laboratory, Richland, WA 99352, USA; 7 Department of Chemistry, Purdue University, 560 Oval Drive, West Lafayette, IN 47907, USA

**Keywords:** Legionella, bacterial effectors, ADP-ribosylation, phosphodiesterase

## Abstract

Ubiquitination regulates many aspects of host immunity and thus is a common target for infectious agents. Recent studies have revealed that members of the SidE effector family of the bacterial pathogen *Legionella pneumophila* attack several small GTPases associated with the endoplasmic reticulum by a novel ubiquitination mechanism that does not require the E1 and E2 enzymes of the host ubiquitination machinery. In this case, ubiquitin is first activated by ADP-ribosylation at Arg_42_ by a mono-ADP-ribosyltransferase activity; the intermediate is then cleaved by a phosphodiesterase activity also residing within SdeA, concomitant with the attachment of ubiquitin to serine residues of substrate proteins via a phosphoribosyl linker. Here we demonstrate that the effect of SidEs is antagonized by SidJ, an effector encoded by a gene situated in the locus coding for three members of the SidE family (SdeC, SdeB and SdeA). SidJ reverses ubiquitination of SidEs-modified substrates by cleaving the phosphodiester bond that links phosphoribosylated ubiquitin to protein substrates. SidJ also displays classical deubiquitinase activity but does not require catalytic cysteine residues. Further, these deubiquitinase activities of SidJ are essential for its role in *L. pneumophila* infection. Finally, the activity of SidJ is required for efficiently reducing the abundance of ubiquitinated Rab33b in infected cells within a few hours after bacterial uptake. Our results establish SidJ as a ubiquitin-deconjugating enzyme that functions to impose temporal regulation on the activity of SidE effectors. SidJ may be important in future studies of signaling cascades mediated by this unique ubiquitination, one that also potentially regulates cellular processes in eukaryotic cells.

## Introduction

The ubiquitin network constitutes one of the most important signaling mechanisms in eukaryotes; post-translational modification by the 76-residue peptide normally is catalyzed by the E1, E2 and E3, three-enzyme cascade, which is tightly regulated by various factors including deubiquitinases that function to remove ubiquitin from modified substrates^1^. Ubiquitination is essential for the execution of many essential cellular processes such as cell cycle, development and immunity^2^. Thus, effective interference with host ubiquitin signaling is critical for the success of many infectious agents.

The study of virulence factors involved in the perturbation of the host ubiquitin network has provided insights into not only the mechanism of infection, but also the mechanism of ubiquitination. For example, the discovery of the HECT family of E3 ligases largely was due to the study of the E6 oncogenic protein from human papillomavirus (HPV)^3^. Similarly, many bacterial virulence factors have been shown to interfere with the ubiquitin network by functioning as E3 ligases or deubiquitinases; such studies have been important for elucidating the mechanism of action and substrate selection of these enzymes^4^.

*Legionella pneumophila*, the etiological agent of Legionnaires' disease, is a ubiquitous bacterial pathogen that exists largely as a parasite of freshwater unicellular eukaryotes such as amoebae^5^. After phagocytosis the bacterium resides in a phagosome called the *Legionella*-containing vacuole (LCV). This pathogen employs similar strategies for the interactions with amoebae or with human alveolar macrophages, resulting in a unique trafficking route that allows the LCV to bypass the endocytic pathway^6^. During this process, the LCV sequentially recruits several host organelles including the endoplasmic reticulum (ER), mitochondria and ribosomes, resulting in the conversion of the phagosome into a compartment with features of the ER^6^. It is within this ER-like compartment that the bacterium extensively replicates. Successful establishment of the LCV permissive for *L. pneumophila* replication requires the Dot/Icm type IV secretion system, which delivers into the host cell hundreds of effectors that modulate various cellular processes such as vesicle trafficking, cell death, autophagy, phospholipid metabolism and ubiquitination, which benefit the bacterium^6^.

Modulation of host ubiquitination pathways by Dot/Icm substrates has emerged as an important theme in the pathogenicity of *L. pneumophila*^7^. Ubiquitinated species are enriched on the LCV and genetic or chemical interference with such associations disturbs bacterial intracellular replication^8^. Among the ∼300 effectors injected into host cells by *L. pneumophila*^9,10,11^, eight appear to possess F-box or U-box domains typical of ubiquitin E3 ligases, and such activity has been demonstrated for LegU1, LegAU13/AnkB, LubX and GobX^12,13,14,15^. Ubiquitin E3 ligase activity catalyzed by mechanisms that differ from the typical members of the HECT or the RING finger families has been documented for SidC, SdcA^16^, which contribute to the enrichment of ubiquitinated proteins on the LCV^16^.

The SidE family (SdeA, SdeB, SdeC and SidE) of effectors are distinguished from the majority of other effectors by their importance for intracellular bacterial replication in the protozoan host *Dictyostelium discoideum*^17,18^. These large proteins appear to contain multiple functional domains that possess distinct biochemical activities involved in ubiquitin signaling. The N-terminal 200-residue domain of all members is a deubiquitinase conferred by a Cys-His-Asp catalytic triad capable of recognizing both ubiquitin and NEDD8^19^. The central domain is an all-in-one ubiquitin ligase that catalyzes ubiquitination of several Rab small GTPases without requiring the E1 and E2 enzymes^20^. In this ubiquitination reaction, ubiquitin is activated by ADP-ribosylation at Arg_42_ at the expense of nicotinamide adenine dinucleotide (NAD) to form ADP-ribosylated ubiquitin (ADPR-Ub)^20^. Activated ubiquitin is attached to serine residues of the substrate via a phospho-ribose bridge formed by a reaction catalyzed by a phosphodiesterase (PDE) domain harbored by the same proteins^21,22^. Members of the SidE family also regulate the morphology of the ER by ubiquitinating reticulon 4 (Rtn4), leading to its oligomerization and enrichment on the bacterial phagosome^22^.

The activity of the SidEs appears to be affected by SidJ (Lpg2155), an effector encoded by a gene situated in the locus coding for some members of the SidE family^23^. SidJ suppresses the yeast toxicity conferred by members of the SidE family, suggesting that it affects the activity of the SidEs^24^. Here we demonstrate that SidJ functions to remove ubiquitin from proteins modified by the SidEs. We also show that SidJ has deubiquitinase activity against ubiquitination induced by the conventional three-enzyme cascade. During bacterial infection, the effects of SidJ do not become apparent until 4 h after uptake, indicating that this effector imposes a temporal regulation on the activity of the SidE family.

## Results

### Residues in three regions of SidJ are important for its ability to suppress the effects of SdeA on eukaryotic cells

**Figure 1:**
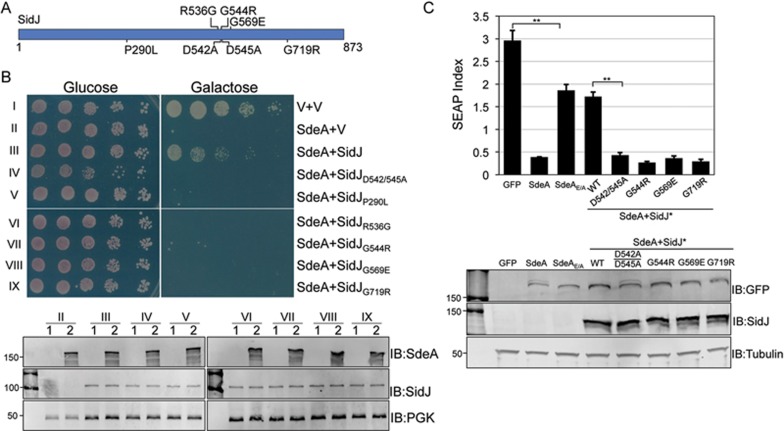
Identification of SidJ substitution mutants unable to suppress SdeA yeast toxicity. **(A**) Distribution of substitution mutations that abolished the ability of SidJ to suppress the yeast toxicity of SdeA. Note the clustering of mutations around the 540th residue of SidJ. (**B)** Yeast strain expressing chromosomally integrated SdeA controlled by a galactose-inducible promoter was transformed with plasmids carrying WT or mutated SidJ controlled by the alcohol dehydrogenase (ADH) promoter. Serially diluted yeast cells were spotted onto glucose or galactose medium. Images were acquired 3 days after incubation at 30 °C. Lower panel showed the expression of SdeA and SidJ, yeast cells grown in medium supplemented with glucose (1) were induced with galactose (2) for 8 h, and the total proteins separated by SDS-PAGE were detected by immunoblotting with antibodies for SdeA or SidJ, respectively. The 3-phosphoglycerate kinase (PGK) was probed as a loading control. **(C)** 293T cells were transfected with the plasmid that directs the expression of the secreted embryonic alkaline phosphatase (SEAP), GFP-SdeA and SidJ or its mutants for 24 h. The activity of SEAP in culture supernatant or the cells was measured to calculate the SEAP index. GFP and GFP-SdeA_E/A_ that expressed the SdeA mutant defective in E_860_ and E_862_, two residues critical for the mono-ADP-ribosyltransferase activity important for the activation of ubiquitin by ADP-ribosylation were used as controls. Lower panel showed the expression of SdeA and SidJ. Cells were lysed and total proteins separated by SDS-PAGE were probed with antibodies against GFP or SidJ; tubulin was probed as a loading control. SidJ^*^, SidJ mutants. Error bars represent s.e. from three independent experiments. ^**^P< 0.01.

Members of the SidE family are toxic to eukaryotic cells such as yeast^24^ in a way that entirely depends on a mono-ADP-ribosyltransferase (mART) motif in the central region of each of these proteins^20^. Mutations in this motif such as those in the SdeA_E/A_ mutant in which E860 and E862 were substituted with alanine completely abolished its activity^20^. Similar to the close proximity seen in other effector pairs with opposite biochemical activity^6^, SidJ is adjacent to SdeA, SdeB and SdeC of the SidE family and is able to suppress SdeA toxicity to yeast^24^. To gain insights into the mechanism of action of SidJ, we isolated substitution mutants defective in their ability to suppress the yeast toxicity of SdeA. To this end, plasmid DNA carrying *sidJ* treated with hydroxylamine^25^ was introduced into a yeast strain expressing SdeA from a galactose-inducible promoter^20^. Transformants unable to grow on inducing (galactose) medium harbor candidate SidJ mutants that have lost the suppressor activity. By screening 200 candidate mutants defective in such activity, we obtained five mutants which still encoded full-length proteins. Sequencing analysis revealed that these mutations (P290L, R536G, G544R, G569E, G719R) mapped onto three regions of SidJ, localized around the 290th, the 540th and the 719th residues, respectively (Figure 1A). Three of these mutations mapped to positions close to D542 and D545, which are critical for the ability of SidJ to rescue the yeast toxicity of SdeA^24^ (Figure 1B). None of these mutations affected the stability of SidJ, but all had lost the ability to suppress yeast toxicity by SdeA (Figure 1B). In addition, these mutants also failed to suppress SdeA-mediated inhibition of the secretion of the secreted embryonic alkaline phosphatase (SEAP) by mammalian cells (Figure 1C). These residues are either critical for the catalytic activity of SidJ or are important for SidJ to maintain its conformation.

SidJ is a member of a two-gene family; its homolog SdjA which shares 52% identity and 60% similarity to SidJ is also a substrate of the Dot/Icm transporter^23^. Importantly, all of the residues critical for the suppressor activity of SidJ are conserved in SdjA. We thus examined whether SdjA has activity similar to that of SidJ. Unexpectedly, although SdjA was expressed, it did not rescue yeast from SdeA toxicity (Supplementary information, Figure S1A), suggesting that these two proteins may have different functions.

### SidJ removes ubiquitin from Rab ubiquitinated by SdeA

**Figure 2:**
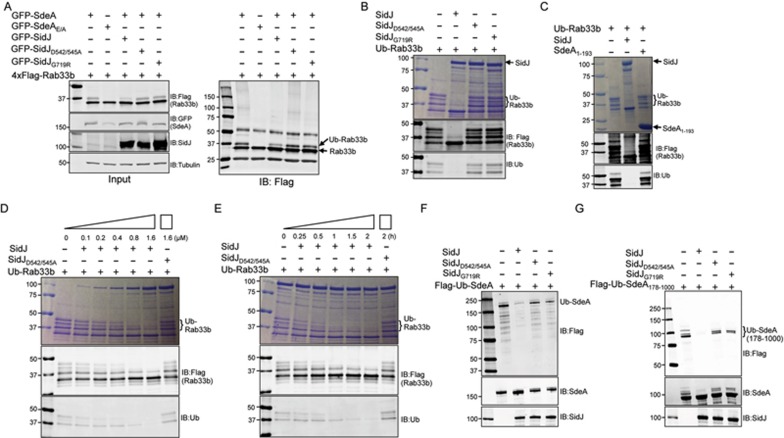
The reversal of SdeA-induced ubiquitination by SidJ. **(A)** SidJ interferes with ubiquitination catalyzed by SdeA in cells. 293T cells were transfected to express Flag-Rab33b together with the indicated combinations of SdeA, SidJ or their mutants. Relevant proteins were probed in total lysate to determine protein expression and tubulin was detected as a loading control (left panel). Immunoprecipitated Flag-Rab33b was probed by immunoblotting with the Flag antibody, ubiquitinated Rab33b (Ub-Rab33b) was assessed by an increase in molecular weight (right panel). Note that wild-type SidJ but not the non-suppressing mutants reduced Rab33b ubiquitination (compare the 4th lane to the 2nd, 5th or the 6th lane). **(B)** The removal of ubiquitin from modified Ub-Rab33b by SidJ. 6 μM of purified Ub-Rab33b was incubated with 1.6 μM of SidJ or its mutants for 2 h and the level of ubiquitination was probed by staining (upper panel) or immunoblotting with antibody specific for the Flag epitope on Rab33b (middle panel) or for ubiquitin (lower panel). Note that mutants SidJ_D542/545A_ and SidJ_G719R_ still have detectable activity indicated by the increase of unmodified Rab33b in reactions containing each of these mutants. **(C)** Ubiquitination induced by SdeA cannot be removed by the conventional deubiquitinase SdeA_1-193_. 6 μM of Ub-Rab33b was incubated with 5 μM of SdeA_1-193_ or 1.6 μM of SidJ for 2 h and activity was evaluated as described in B. **(D**, **E)** Dose- and time-dependent deubiquitination of Rab33b by SidJ. A series of reactions containing Ub-Rab33b and SidJ were established. After 2 h incubation, samples separated by SDS-PAGE were evaluated for Ub-Rab33b as described in B **(D)**. For time-dependent reactions, a series of identical samples containing 6 μM Ub-Rab33b and 1.6 μM SidJ were established and each was terminated at the indicated time durations **(E)**. (**F**, **G)** The removal of ubiquitin from SdeA and its deletion mutant SdeA_178-1 000_ by SidJ. 6 μM of SdeA **(F)** or SdeA_178-1 000_ **(G)** modified by Flag-ubiquitin was incubated with 1.6 μM of SidJ or its mutants for 2 h. Ubiquitinated SdeA and SdeA_178-1 000_ were probed by immunoblotting with a Flag-specific antibody (top panels). SdeA, SdeA_178-1 000_, SidJ and its mutants used in the reactions were probed by immunoblotting (middle and lower panels). Note the reduction or disappearance of ubiquitin signals associated with SdeA or SdeA_178-1 000_ after SidJ treatment. The two SidJ mutants have markedly reduced activity. Similar results were obtained in at least three independent experiments in all panels.

To determine the mechanism of action of SidJ, we co-expressed this protein in mammalian cells with SdeA and Rab33b, one of its substrates^20^. Consistent with an earlier observation^20^, SdeA induced ubiquitination of Rab33b (Figure 2A). Importantly, the intensity of the band representing modified Rab33b decreased in cells co-expressing SidJ. Consistent with their inability to suppress toxicity in yeast, mutants SidJ_D542/545A_ and SidJ_G719R_ were less effective in reducing Rab33b ubiquitination induced by SdeA (Figure 2A). The effects of SidJ on Rab33b ubiquitination in cells may be a result of its ability to inhibit the enzymatic activity of SdeA or to reverse the modification of its substrates. To distinguish between these two possibilities, we incubated ubiquitinated Rab33b (Ub-Rab33b) with recombinant SidJ or its mutants. Under our experimental conditions, in reactions containing 6 μM of Ub-Rab33b, 1.6 μM of SidJ was able to completely remove the ubiquitin moiety from the substrate within 2 h at 37 °C as indicated by the reversal of gel-shifted protein bands of higher molecular weight and an increase of unmodified Rab33b (Figure 2B). Consistent with the phenotypes of suppression of toxicity and inhibition of secretion, both SidJ_D542/545A_ and SidJ_G719R_ did not function well in releasing ubiquitin from Ub-Rab33b (Figure 2B). The N-terminal end of SdeA harbors a deubiquitinase domain (SdeA_Dub_) that functions to remove ubiquitin from proteins modified by the canonical pathway^19^. SdeA_Dub_ did not detectably deubiquitinate Ub-Rab33b (Figure 2C). This is consistent with our earlier finding that SdeA_Dub_ does not interfere with its ubiquitin ligase activity^20^.

Deubiquitination of Ub-Rab33b by SidJ was both dose- and time-dependent. Under the reaction condition employed, 1.6 μM of SidJ was able to almost completely remove ubiquitin from 6 μM of Ub-Rab33b in 2 h (Figure 2D). Similarly, in reactions containing 1.6 μM of SidJ and 6 μM of Ub-Rab33b, deubiquitination became apparent within 30 min, and 2 h incubation was required to completely remove ubiquitin from the modified substrate (Figure 2E). Importantly, SidJ also removed ubiquitin from self-modified SdeA and from its truncation mutant SdeA_178-1 000_ lacking the Dub domain. In contrast, the two non-suppressing mutants SidJ_D542/545A_ and SidJ_G719R_ were less active in deconjugating Ub-SdeA and Ub-SdeA_178-1 000_ (Figure 2F-2G).

The ability of SidJ to remove ubiquitin from Ub-Rab33b prompted us to determine whether this protein interacts with the modifier. Under the conditions employed, no protein complex was detected in reactions containing GST-ubiquitin and SidJ (Supplementary information, Figure S2B). Thus, these results suggest that SidJ either does not bind ubiquitin or such binding, if it occurs, cannot be detected by affinity pull-down.

We attempted to further define the domain structure of this protein by deletion analysis. Removal of 100 amino acids from the N-terminal end of SidJ gave rise to a mutant that retained the ability to antagonize SdeA-induced ubiquitination of Rab33b; further deletion of an additional 100 residues completely abolished the activity (Supplementary information, Figure S2A). Deletion of 100, 200, 300 or 400 amino acids from the C-terminus were all inactive (Supplementary information, Figure S2A). Our deletion analyses confirm the importance of residues between 200 and 720 for SidJ activity (Figure 1); these results also suggest that multiple regions of SidJ participate in the formation of its functionally important sites, presumably through catalytic pockets.

We also examined the activity of SdjA on Ub-Rab33b. Consistent with the observation that SdjA failed to rescue the inhibition of yeast growth, it did not detectably affect the ubiquitination of Rab33b when co-expressed with the ligase in mammalian cells (Supplementary information, Figure S1B). Furthermore, recombinant SdjA was unable to deconjugate ubiquitin from Ub-Rab33b modified by SdeA (Supplementary information, Figure S1C).

### SidJ has canonical deubiquitinase (Dub) activity

**Figure 3:**
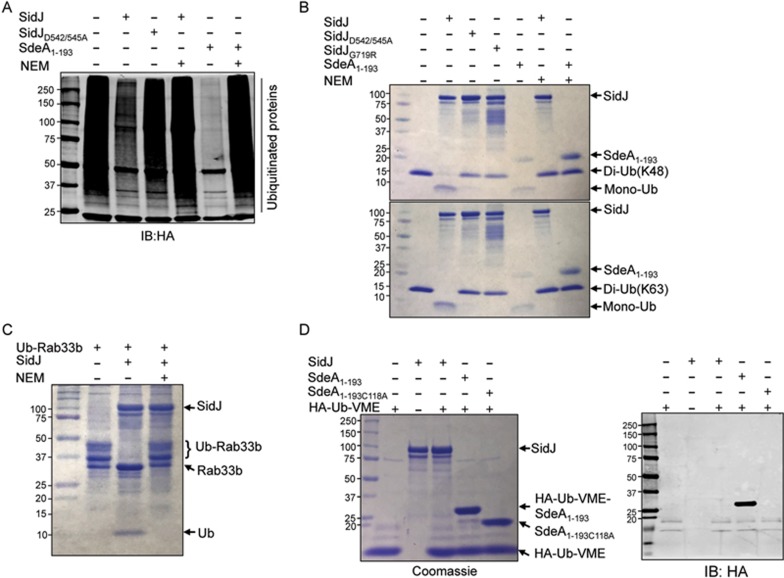
The activity of SidJ against ubiquitinated proteins and diubiquitins. **(A)** 2 μM of recombinant SidJ or its mutants was added to ubiquitinated proteins purified from 293T cells transfected to express HA-Ub and the reactions were allowed to proceed for 1 h before SDS-PAGE separation. The level of ubiquitinated proteins was evaluated by immunoblotting with an HA-specific antibody. SdeA_1-193_, the Dub domain of SdeA was included as a control. The alkylating reagent N-ethylmaleimide (NEM) was added to 2 mM. Note that NEM effectively inhibited the activity of both SidJ (5th lane) and SdeA_Dub_ (7th lane). **(B)** The cleavage of isopeptide bond by SidJ. 3 μM of SidJ, its mutants or 5 μM of SdeA_Dub_ was incubated with diubiquitin of K48 (upper panel) or K63 (lower panel) linkage for 2 h and the products of the reactions were detected by Coomassie staining. Note the complete digestion by SidJ and SdeA_Dub_ and their sensitivity to NEM. Both SidJ_D542/545A_ and SidJ_G719R_ retained detectable activity (compare the remaining diubiquitin in lanes 4 and 5 to the control or the reaction containing NEM). **(C)** The removal of ubiquitin from Ub-Rab33b by SidJ is sensitive to the alkylating reagent NEM. 2 mM NEM was added to reactions used to examine the removal of ubiquitin from Ub-Rab33b by SidJ and ubiquitination status of Rab33b was detected by Coomassie staining. Note the release of ubiquitin by SidJ from Ub-Rab33b. **(D)** Detection of potential reactive cysteine residues in SidJ by the HA-Ub-VME probe. SidJ, SdeA_1-193_ or SdeA_1-193C118A_ was mixed with the probe for 1 h and the formation of the ubiquitin adduct was detected by staining (left panel) or by immunoblotting with an HA-specific antibody (right panel). Note that SdeA_1-193_ but not SidJ or SdeA_1-193C118A_ reacts with the probe to form a complex with higher molecular weight. Similar results were obtained in at least two independent experiments in all panels.

To examine whether SidJ has activity against proteins ubiquitinated by the canonical pathway, we incubated recombinant SidJ with ubiquitinated proteins purified from mammalian cells expressing HA-Ub^19^. The addition of recombinant SidJ to reactions containing these proteins led to the removal of ubiquitin (Figure 3A). In this *in vitro* reaction, the efficiency of deubiquitination catalyzed by SidJ against ubiquitinated proteins modified by the conventional mechanism was considerably lower than that of SdeA_Dub_. The mutant SidJ_D542/545A_, which is incapable of suppressing SdeA toxicity, exhibited markedly lower activity (Figure 3A). These results suggest that SidJ possesses canonical deubiquitinase activity.

To determine whether SidJ prefers specific linkage types formed by canonical ubiquitination, we examined its ability to cleave diubiquitin formed by K48 and K63 linkages, respectively. SidJ completely hydrolyzed these substrates in a 2 h incubation; the activity of both mutants SidJ_G719R_ and SidJ_D542/545A_ was greatly reduced (Figure 3B). In addition, SidJ was able to completely cleave diubiquitin linked by K11 but only partially cleave that linked by K33 under this experimental condition (Supplementary information, Figure S3A). We further examined the potential linkage type preference of SidJ by shortening the reaction time. After 10 min incubation, more than 60% of the K63-linked diubiquitin was cleaved. However, less than 10% cleavage occurred in reactions containing K11- or K48-linked diubiquitin. In either case, further incubation led to more extensive cleavage of diubiquitin (Supplementary information, Figure S3B-S3C). Thus, SidJ can recognize the isopeptide bond in K11-, K33-, K48- and K63-linked diubiquitins with a preference for the K63-linked bond.

Because deubiquitination is often catalyzed by the Cys-His-Asp catalytic triad whose cysteine residue is critical for activity^26^, we determined the effects of the alkylating reagent N-ethylmaleimide (NEM), a known inhibitor of cysteine proteases^27^ on the activity of SidJ. The addition of NEM completely abolished the activity of SidJ against diubiquitins and cellular proteins ubiquitinated by the canonical mechanism (Figure 3A-3B). In controls, SdeA_Dub_ effectively cleaved these diubiquitins and was also sensitive to the alkylating reagent^19^ (Figure 3B). When Ub-Rab33b was used as substrate, SidJ effectively removed ubiquitin from the modified protein, leading to the accumulation of free ubiquitin. Intriguingly, inclusion of NEM in the reactions also completely abolished such activity (Figure 3C). Similar to its inability to reverse SdeA-induced ubiquitination on Rabb33b, SdjA did not remove ubiquitin from proteins modified by conventional ubiquitination (Supplementary information, Figure S1D).

The sensitivity of SidJ to NEM suggested the involvement of one or more cysteine residues in catalysis. We thus created mutants of SidJ that lacked each of the three cysteine residues and examined their activity in several different assays. When transformed into the yeast strain inducibly expressing SdeA, these mutants still effectively suppressed the toxicity of the ubiquitin ligase (Supplementary information, Figure S4A). Consistent with this observation, these mutants were still active in counteracting the ability of SdeA to ubiquitinate Rab33b when co-expressed with SdeA in mammalian cells, albeit at a detectably lower level (Supplementary information, Figure S4B). In agreement with the partial loss of activity seen in the co-transfection experiments, the cysteine mutants were able to remove ubiquitin from Ub-Rab33b at a low efficiency (Supplementary information, Figure S4C). Similarly, these mutants were still active in removing ubiquitin from proteins modified by the canonical ubiquitination (Supplementary information, Figure S4D). Furthermore, these mutants were still active in hydrolyzing K63-linked diubiquitin but at a considerably lower efficiency (Supplementary information, Figure S4E).

Overall, these results indicate that none of the three cysteine residues is essential for the enzymatic activity of SidJ. These residues may contribute to the structure and stability of the protein, features that are important but not essential for the activity of the enzyme.

We further examined the potential involvement of the cysteine residues in the activity of SidJ using the HA-Ub-VME probe, which is designed for the detection of active canonical Dub domains containing a catalytic cysteine^28^, but there were no detectable covalent adducts formed (Figure 3D). In control experiments, the SdeA_Dub_ domain displayed a cysteine-dependent reactivity characterized by an approximate 8 kDa molecular weight shift resulting from the formation of a ubiquitin adduct (Figure 3D). Thus, the deubiquitination reaction catalyzed by SidJ against canonical ubiquitination is achieved by a mechanism different from those used by classic Dubs^26^. The inability to react with HA-Ub-VME also supports the notion that cysteine residues are not directly involved in the catalysis by SidJ.

We also examined the effects of several protease inhibitors on the activity of SidJ. At concentrations suggested by the manufacturer, none of the four inhibitors, including 4-(2-aminoethyl)benzenesulfonyl fluoride (AEBSF, an irreversible serine protease inhibitor), Bestatin (an aminopeptidase inhibitor), Pepstatin A (an aspartyl proteases inhibitor) and E-64 (a cysteine protease inhibitor) detectably blocked the activity of SidJ against Ub-Rab33b (Supplementary information, Figure S5A). Thus, the cleavage of chemical bonds by SidJ is catalyzed by a mechanism different from proteases sensitive to these inhibitors.

Finally, we examined the substrate specificity of SidJ using neddylated proteins obtained from transfected cells expressing Flag-NEDD8. In controls, the Dub domain of SdeA deconjugated NEDD8 modification (Supplementary information, Figure S5B), which was similar to previous results^19^. In contrast, SidJ did not detectably remove NEDD8 from modified proteins (Supplementary information, Figure S5B).

### SidJ removes ubiquitin from substrates of SdeA by cleaving the phosphodiester bond

**Figure 4:**
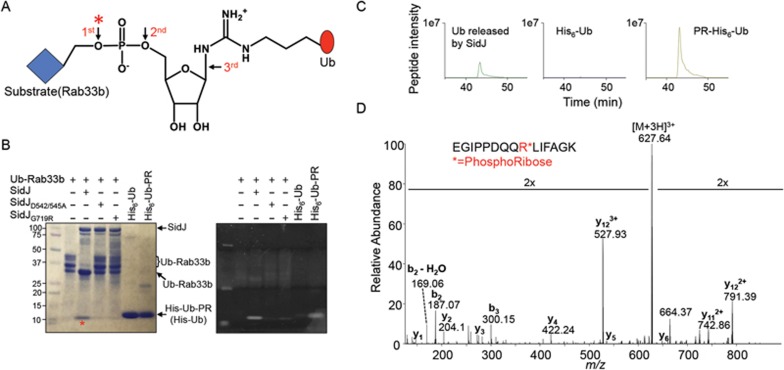
SidJ deconjugates ubiquitin by cleaving the phosphodiester bond that links phosphoribosylated ubiquitin to the substrate. **(A)** The chemical bonds that link ubiquitin to its substrate. The three arrows indicate the site of cleavage that can lead to the release of ubiquitin from the substrate. ^*^ indicates the cleavage site of SidJ. **(B)** The ubiquitin species released from Ub-Rab33b by SidJ contains phosphate. 6 μM of Ub-Rab33b was incubated with 1.6 μM of SidJ or its mutants; samples resolved by SDS-PAGE were detected by Coomassie staining (left panel) or by Pro Q diamond phosphoprotein stain (right panel). PR-Ub and His-Ub used for the modification were included as controls. The red ^*^ denotes the protein band used for mass spectrometric analysis. **(C)** Mass spectrometric analysis of the ubiquitin species released from Rab33b by SidJ. The extracted ion chromatograms at m/z 627.64 correspond to the triply-charged peptide from ubiquitin -E_34_GIPPDQQRLIFAGK_48_- containing the phosphoribose moiety. Note that the signal was present in PR-Ub and ubiquitin species released from Ub-Rab33b by SidJ but not in free ubiquitin. **(D)** Ultraviolet photon dissociation (UVPD) mass spectrum of the peptide -E_34_GIPPDQQRLIFAGK_48_- showing the attachment of the phosphoribose moiety to Arg_42_ of ubiquitin. Similar results were obtained in three independent experiments in each panel.

Ubiquitination induced by SdeA occurs by attaching phosphoribosylated ubiquitin (PR-Ub) via a phosphodiester bond to serine residues of substrates such as Rab33b^21^. Cleaving one of the two phosphodiester bonds or the N-glycosidic bond that directly links the ribose moiety to Arg_42_ of ubiquitin will release the ubiquitin moiety (Figure 4A). To determine the mechanism of SidJ-mediated deubiquitination from substrates of SidEs, we incubated Ub-Rab33b with SidJ and detected products by staining with Coomassie brilliant blue and Pro-Q Diamond phosphoprotein stain, respectively. Reaction with SidJ but not SidJ_D542/545A_ or SidJ_G719R_ led to the accumulation of ubiquitin species that migrated in a way similar to PR-Ub and the ubiquitin molecule (Figure 4B, left panel). Importantly, similar to PR-Ub produced by SdeA together with ubiquitin and NAD^21^, the products released from Ub-Rab33b by SidJ were detected by the Pro-Q Diamond phosphoprotein stain (Figure 4B, right panel), suggesting that SidJ cleaves the phosphodiester bond that links PR-Ub to its substrate.

To determine more unequivocally the site of cleavage, we performed mass spectrometric analysis of the ubiquitin species released by SidJ from Ub-Rab33b. We chose to perform the analysis using ultraviolet photon dissociation (UVPD) because it is selective for the peptide bond, without strong fragmentation of often labile post-translational modifications, thus providing a more informative tandem mass spectrum compared to collision-induced dissociation^29^. A phosphoribose moiety attached to Arg_42_ of ubiquitin was detected in the tryptic peptide – E_34_GIPPDQQRLIFAGK_48_ (Figure 4C-4D). If SidJ-mediated cleavage occurs at the second site or simultaneously at both the first and third sites, ribosylated ubiquitin and free unmodified ubiquitin should be produced. Yet, neither molecule was detected in the released ubiquitin species (Figures 4A and Supplementary information, Figure S6). These results establish SidJ as a phosphodiesterase that specifically recognizes and cleaves the phosphodiester bond that links PR-Ub to its substrate (Figure 4A, first arrow).

The observation that SidJ cleaves two different chemical bonds prompted us to examine whether SidJ prefers phosphoribosylated Ub linkages over isopeptide bonds. In reactions containing SidJ and equimolar K63-linked diubiquitin and Ub-Rab33b, a 15 min incubation led to complete hydrolysis of the diubiquitin. However, deubiquitination from Ub-Rab33b was minimal (Supplementary information, Figure S5C). Thus, under our experimental conditions, SidJ displayed a preference for K63-linked diubiquitin over Ub-Rab33b.

### The phosphodiesterase activity of SidJ is important for its role in bacterial infection

**Figure 5:**
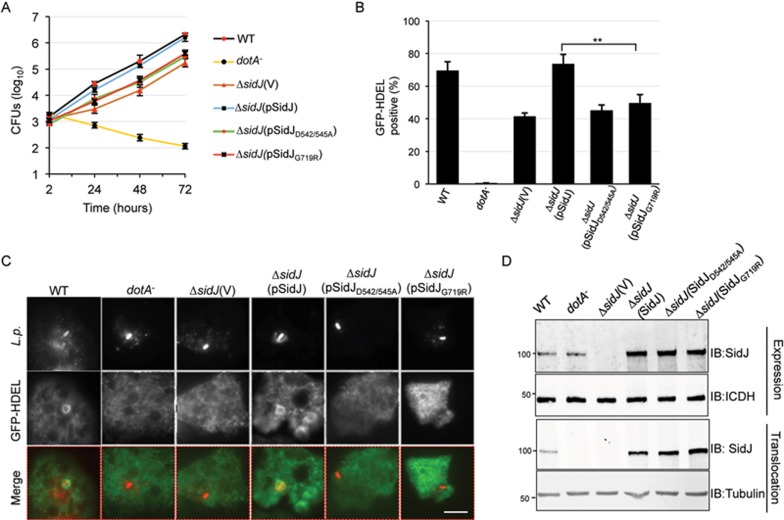
The phosphodiestrase activity of SidJ is important for its role in bacterial infection. **(A)** The enzymatic activity of SidJ is critical for the role of SidJ in intracellular bacterial replication. Mouse bone marrow-derived macrophages were infected with the indicated bacterial strains and the growth of the bacteria was monitored by determining the colony-forming units (CFUs) at the indicated time points. Results shown are one representative of three independent experiments done in triplicate. Error bars, standard error (s.e.). **(B)** The deubiquitinase activity of SidJ is important for efficient recruitment of ER proteins to the bacterial phagosome. A *D. discoideum* strain expressing the HDEL-GFP marker was infected with the indicated bacterial strains for 2 h. Fixed samples were differentially stained for extracellular and intracellular *L. pneumophila*. The association of HDEL-GFP with the vacuoles was scored under a fluorescence microscope. Data showed were from three independent experiments done in triplicate. Error bars, s.e. ^**^P< 0.01. **(C)** Representative images displaying the enrichment of HDEL-GFP signals on the LCV for infection with the relevant bacterial strains. Images acquired with an Olympus IX-81 fluorescence microscope were pseudocolored with the IPLab software package. Bar, 6 μm. **(D)** The expression and translocation of the SidJ mutants. Expression of SidJ and its mutants in *L. pneumophila* strains used for infection was probed with SidJ-specific antibodies (upper panel). Translocated protein was similarly detected in lysates of infected U937 cells prepared with saponin (lower panel). The host protein tubulin was probed as a loading control. Similar results were obtained in three independent experiments.

SidJ is required for optimal intracellular growth of *L. pneumophila* in bone marrow-derived macrophages and for efficient recruitment of ER resident proteins such as calnexin to the bacterial phagosome^23^. Having identified the residues critical for its ubiquitin deconjugation activity, we examined the ability of two mutants defective in such activity to complement the Δ*sidJ* mutant. Expression of wild-type SidJ fully rescued the defect in intracellular replication (Figure 5A). In contrast, neither SidJ_D542/545A_ nor SidJ_G719R_ complemented the phenotype associated with the deletion mutant (Figure 5A). Similarly, expression of SidJ, but neither of these mutants, allowed the Δ*sidJ* mutant to efficiently recruit the GFP-HDEL ER marker to the bacterial phagosome in *D. discoideum* 2 h post bacterial uptake (Figure 5B-5C). Since both mutants were expressed and translocated at levels comparable to that of wild type (Figure 5D), the inability to complement the phenotype must be due to the loss of enzymatic activity.

### The cellular localization of SdeA is not affected by self-ubiquitination or by SidJ

Members of the SidE family undergo self-ubiquitination, the biological significance of which is unclear^20^. SidJ had been shown to promote the loss of SidE family proteins from the LCV at later times of infection^24^. The discovery of the deubiquitination activity of SidJ prompted us to examine whether self-modification has a role in the cellular localization of SdeA.

**Figure 6:**
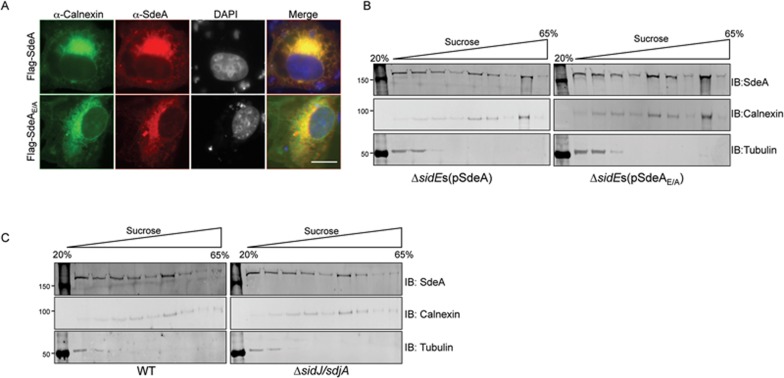
The cellular localization of SdeA is not affected by its self-ubiquitination activity. **(A)** COS-1 cells were transfected to express Flag-SdeA or Flag-SdeA_E/A_ for 24 h. Permeabilized fixed cells were sequentially immunostained with antibodies specific for the ER resident protein calnexin and SdeA; secondary antibodies conjugated to fluorescein and Texas red were used to label calnexin and SdeA, respectively. Note that in both cases, the red fluorescence signals indicative of SdeA co-localized well with those of calnexin. Images were acquired with an Olympus IX-81 fluorescence microscope and were pseudocolored with the IPlab software. Bar, 5 μm. **(B)** Cellular localization of SdeA or SdeA_E/A_ during bacterial infection. Macrophages infected with *L. pneumophila* strains expressing SdeA or the SdeA_E/A_ mutant for 2 h were used to determine the cellular localization of translocated SdeA by biochemical fractionation. The ER resident protein calnexin and the cytosolic protein tubulin were used as references. Note that SdeA co-fractionated with calnexin regardless of its ubiquitin ligase activity. Similar results were obtained from three independent experiments. **(C)** SidJ does not affect the cellular localization of SdeA during bacterial infection. Macrophages infected with wild-type *L. pneumophila* or a mutant lacking *sidJ* and *sdjA* for 2 h were used to determine SdeA distribution in cells by fractionation with sucrose gradient centrifugation. Note that in both samples, SdeA co-fractionated with the ER resident protein calnexin. Similar results were obtained in three independent experiments.

COS1 cells transfected to express Flag-tagged SdeA or SdeA_E/A_ were immunostained with antibodies specific for SdeA and the ER resident protein calnexin, respectively. Expression of SdeA condensed the ER, and the fluorescence signal of SdeA detected by our anti-SdeA antibodies extensively co-localized with that of calnexin (Figure 6A). The SdeA_E/A_ mutant defective in self-ubiquitination^20^ has largely lost the ability to induce ER morphological changes but it still extensively co-localized with calnexin (Figure 6A). In both cases, such co-localization was seen in all cells expressing SdeA or SdeA_E/A_. Thus, self-ubiquitination of SdeA does not alter its cellular localization.

We further investigated the organelle targeting of SdeA under more physiologically relevant conditions by discontinuous sucrose gradient centrifugation of cells infected by *L. pneumophila* and found that SdeA and SdeA_E/A_ distributed indistinguishably in infected macrophages (Figure 6B). Together, these results indicate that the localization of SdeA in cells infected by *L. pneumophila* is independent of its ubiquitin ligase activity. This indicates that self-ubiquitination does not change the cellular localization of this effector.

To determine the role of SidJ in the cellular localization of SdeA, we compared the fractionation pattern of SdeA translocated into host cells by wild-type *L. pneumophila* or a strain defective in *sidJ*. In both cases, SdeA co-fractionated with the ER resident protein calnexin (Figure 6C). Thus, SidJ activity did not impact the cellular distribution of SdeA. We also determined the cellular localization of SidJ by expressing RFP-SidJ with GFP or GFP- fused to the *Legionella* phosphoinositide 3-phosphatase SidF, an effector known to localize to the ER^30,31^. RFP signals representing SidJ co-localized extensively with GFP-SidF (Supplementary information, Figure S7). Co-localization with GFP in the cytosol was also detectable (Supplementary information, Figure S7). Thus, in host cells, SidJ localizes to both the ER and the cytoplasm, with a preference for the ER.

We have also attempted to detect the effects of SidJ on the cellular localization of SidEs during *L. pneumophila* infection by immunostaining. Despite extensive efforts with purified antibodies specific for SdeA or SdeC under the described experimental procedures^18,24^ and a number of conditions modified from these procedures, we were unable to detect the association of SidEs with bacterial phagosomes in infected cells or isolated LCVs. This was despite the use of antibodies against SdeA or SdeC that were able to specifically detect endogenous proteins in immunoblotting when used at dilutions as high as 1:100 000 (Supplementary information, Figure S8); these antibodies also robustly detected proteins ectopically expressed in mammalian cells by immunostaining (Figure 6A), suggestive of a comparable sensitivity to those used in earlier studies^18,24^.

### SidJ temporally regulates the effects of the SidE family during bacterial infection

**Figure 7:**
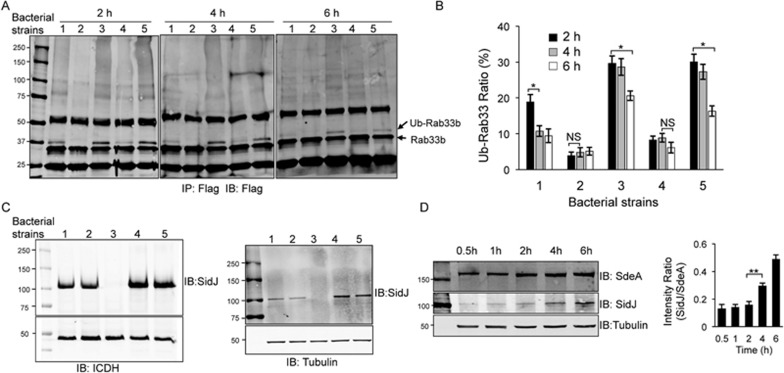
SidJ is required for efficient removal of ubiquitin from Rab33b several hours post bacterial uptake. **(A)** HEK293 cells transfected to express Flag-Rab33b were infected with relevant bacterial strains. At the indicated time points, lysates of infected cells were subjected to immunoprecipitation with Flag antibody and the products were probed by a Flag-specific antibody. Ubiquitinated Flag-Rab33b was distinguished by an increase in molecular weight. Bacterial strains used: 1, wild type; 2, Lp02Δ*sidE* (lacking all members of the *sidE* family); 3, Lp02Δ*sidJ/sdjA*; 4, Lp02 Lp02Δ*sidJ/sdjA*(pSidJ); 5, Lp02Δ*sidJ/sdjA*(pSidJ_D542/545A_). **(B)** Quantitation of ubiquitinated Flag-Rab33b. The ratio of Ub-Flag-Rab33b was calculated by dividing the intensity of the band corresponding to Ub-Flag-Rab33b by that of Flag-Rab33b obtained by Image J. Results shown were from three independent experiments. Scale bars represent s.e. NS, not significant. ^*^P< 0.05. **(C)** Expression and translocation of SidJ or its mutant by *L. pneumophila*. Lysates of bacteria (left panel) or U937 cells infected with the relevant bacterial strains for 2 h (right panel) were probed for SidJ; the bacterial metabolic enzyme isocitrate dehydrogenase (ICDH) and the host protein tubulin were probed as loading controls for bacterial and host cells, respectively. **(D)** The levels of translocated SidJ and SdeA in the first 6 h of bacterial infection. U937 cells infected with wild-type *L. pneumophila* were lysed with saponin at the indicated time points and the presence of SidJ and SdeA was probed by immunoblotting. The host protein tubulin was detected as a loading control (lower panel). The intensity of bands of protein of interest in three independent experiments was measured by Image J and the ratios of SidJ to SdeA were plotted for each time point examined (right panel). Bars represent s.e. ^**^P< 0.01.

Since SidJ does not appear to alter the cellular localization of SdeA, we considered the possibility that it has a role in the temporal regulation of these ubiquitin ligases. HEK293 cells expressing 4× Flag-Rab33b infected with pertinent *L. pneumophila* strains were examined for the extent of Rab33b ubiquitination at different times after bacterial uptake. Similar to earlier results^20^, infection with wild-type bacteria but not the Δ*sidEs* mutant resulted in ubiquitination of Rab33b 2 h after bacterial uptake (Figure 7A-7B). Consistent with the deubiquitinase activity of SidJ, the amount of Ub-Rab33b in cells infected with a strain lacking *sidJ* was considerably higher, as was also the case for infections with the mutant complemented with the enzymatically inactive SidJ (Figure 7A-7B, strains 3 and 5). Of note, these mutations affect neither the expression nor the translocation of SidJ into infected cells at 6 h post infection (Figure 7C), indicating that the persistence of Ub-Rab33b in cells infected with these strains was due to the loss of enzymatic activity.

At 4 h post infection, the level of Ub-Rab33b became lower in samples infected with the wild type. When the infection was extended to 6 h, the Ub-Rab33b in these samples became barely detectable (Figure 7A-7B, strain 1). Consistent with the notion that SidJ functions to remove ubiquitin from the substrate, in infections with the complementation strain in which SidJ was expressed from a multicopy plasmid, the level of Ub-Rab33b was considerably lower even at 2 h postinfection and became further reduced at 4 h (Figure 7A-7B, strain 4).

Since the relative abundance of two proteins with opposite biochemical activities can influence the status of modification of the target molecule, we examined the ratio between SidJ and SdeA over time in cells infected with *L. pneumophila*. In the first 2 h, translocated SdeA increased but appeared to become constant 2 h after bacterial uptake. In contrast, the amount of translocated SidJ increased within the duration of the entire experiment duration (Figure 7D, left panel). As a result, the ratio of SidJ to SdeA increased as infection proceeded beyond 4 h postinfection (Figure 7D, right panel). Clearly, this change would allow the activity of SidJ to overwhelm that of the SidEs at later phases of infection, thus contributing to the reduction of ubiquitinated substrates.

Intriguingly, in cells infected with bacterial strains lacking SidJ activity, the levels of Ub-Rab33b also declined as the infection proceeded, especially at 6 h postinfection (Figure 7A-7B, strains 3 and 5). This decrease may result from host enzymes with activity similar to that of SidJ. Taken together, these results establish that SidJ temporally regulates ubiquitination imposed by SidE proteins during *L. pneumophila* infection.

## Discussion

The study of the SidE family effectors of *L. pneumophila* has revealed a novel mechanism of ubiquitination that does not require the E1 and E2 enzymes. In this two-step reaction, ubiquitin is first activated by ADP-ribosylation at residue Arg_42_ by an mART motif in SdeA^20^. Then a PDE domain upstream of the mART catalyzes the subsequent attack of a substrate serine residue (or water) with ADPR-Ub at the β phosphate of the ADP group, resulting in the attachment of phosphoribosylated ubiquitin to serine residues of substrates (or the production of PR-Ub)^21^. Our results demonstrate that this unique ubiquitination can be reversed by a specific enzyme, which is a functional equivalent of deubiquitinases involved in regulating signaling cascades controlled by the canonical ubiquitination mechanism^26^.

The concentration of translocated SidJ in infected cells during *L. pneumophila* infection is conceivably much lower than that used in *in vitro* experiments. Yet, it is able to effectively antagonize the effect of all four members of the SidE family. The requirement of a higher concentration of SidJ to deconjugate ubiquitin from Ub-Rab33b in *in vitro* experiments can very likely be attributed to factors such as low protein stability, non-optimal reaction conditions and the incorrect folding of the protein after purification, or a combination of these factors.

We found that SidJ cleaves both the phosphodiester bond and the isopeptide bond (canonical Dub activity) by a mechanism that differs from those attributed to previously described enzymes. Although the cleavage of these two chemical bonds seems to require a common set of residues in SidJ, it is not clear whether such cleavage is carried out by a single catalytic motif or by two independent motifs. Cysteine residues do not appear to be critical for the catalytic activity of SidJ. First, SidJ mutants lacking one of the three cysteine residues are still able to effectively suppress the yeast toxicity of SdeA. Second, co-expression of these mutants with SdeA still led to reduction in SdeA-induced ubiquitination of Rab33b. Yet, recombinant proteins produced by these mutants displayed reduced ability to deconjugate ubiquitin from Ub-Rab33b and in hydrolyzing diubiquitins, suggesting an accessory role of these residues in the activity of SidJ. This may explain the strong inhibitory effects of the alkylating agent NEM capable of simultaneous inactivation of these cysteine residues. Alternatively, alkylation of these cysteine residues may cause steric hindrance of the formation of the catalytic pocket(s). This possibility is especially relevant for Cys_646_, which localizes in the region that harbors many residues important for the activity of SidJ (Figure 1A). The exact roles played by these cysteine residues in the activity of SidJ will be better understood by future studies based on detailed structural information.

In contrast to the observation that members of *L. pneumophila* effector families with high levels of homology, such as those in the SidC and SidE families, possess similar biochemical activity^16,20^, SdjA, the homolog of SidJ did not exhibit detectable activity for proteins ubiquitinated by either SdeA or via the conventional mechanism (Supplementary information, Figure S1). Interestingly, all of the residues known to be important for the activity of SidJ are conserved in SdjA. Whether SdjA is involved in the regulation of SidEs or for ubiquitin signaling remains to be determined.

Members of the SidE family are toxic to yeast and mammalian cells^24^. On the basis of the ability of PR-Ub to inhibit ubiquitination catalyzed by the classical three-enzyme cascade, Bhogaraju *et al*. proposed that the modified Ub contributes to cytotoxicity and that SidJ overcomes the lethality by inactivating SidEs in the early stages of infection^21^. Yet, in infections with wild-type bacteria, the modification of Rab33b was more extensive in early stages (Figure 7), which argues against the inhibition of SidEs activity by SidJ. Furthermore, a SdeC mutant unable to ubiquitinate but still toxic to eukaryotic cells failed to complement the defect in intracellular growth of the *sidEs* mutant^22^, indicating that host cell toxicity *per se* is not sufficient to support bacterial virulence. It is important to note that the cytotoxicity of PR-Ub was observed only when SdeA was ectopically expressed. Cells infected with *L. pneumophila* are more resistant to cell death stimuli and do not exhibit the cytotoxicity phenotype^30,32,33^. Furthermore, under physiological conditions, it is unlikely that PR-Ub accumulates to levels sufficient to inhibit the canonical ubiquitination machinery due to the presence of the substrates. Ubiquitination by SidEs appears to inhibit the activity of its targets such as Rab33b^20^; it is possible these effectors also target other proteins important for essential host processes, which accounts for the toxicity seen in ectopic expression. Nevertheless, SidJ may contribute to the accumulation of PR-Ub in infected cells via its phosphodiesterase activity on modified substrates such as Rab33b. The lack of detectable effects in infected cells may be attributed to low levels of PR-Ub during infection. Alternatively, PR-Ub may be further converted into free ubiquitin and phosphoribose by enzymes from the host and/or the pathogen.

Based on the observation that SidJ is required for the disappearance of proteins of the SidE family from the LCV detected by immunostaining, Jeong *et al*.^24^ proposed that SidJ spatially regulates the activity of these Ub ligases. The identification of the enzymatic activity of these effectors has allowed us to more carefully examine this model. Since the SidEs undergo extensive self-ubiquitination, it is possible that this modification allows them to be associated with specific organelles in the host cell and that SidJ alters such targeting by removing one or more of the ubiquitin moieties. However, the ubiquitin ligase activity of SdeA did not play a role in its cellular localization nor does the activity of SidJ since SdeA fractionated identically between cells infected with the Δ*sidJ* mutant and those with wild-type bacteria (Figure 6). For unknown reasons, we were unable to detect the association of SidEs with the LCV despite extensive efforts with antibodies specific for SdeA or SdeC following a previously described procedure^18,24^.

Ectopic expression of SdeA has been shown to lead to the formation of punctate foci and severe disruption of the Golgi apparatus, both of which can be relieved by co-expressing SidJ^24^. This reversal by SidJ very likely is due to its deubiquitinase activity, as was SidJ's suppression of the inhibition of host secretion imposed by SdeA (Figure 1C). These results are reminiscent of the activity of Lem3, the dephosphorylcholinase that suppresses the effects induced by the phosphorylcholine transferase AnkX in eukaryotic cells^34^. For example, Lem3 effectively suppresses the severe Golgi structure disruption caused by AnkX^34^.

At least two lines of evidence support the conclusion that the role of SidJ is to impose temporal regulation on the activity of the SidE family. First, within hours after bacterial uptake, SidJ is required for efficient removal of ubiquitin from Ub-Rab33b, the substrate of SidEs (Figure 7). Second, the ratio between SidJ and SidEs becomes higher at later times after bacterial uptake (Figure 7), which allows the activity of SidJ to become dominant, converting substrates of SdeA to their unmodified form.

Temporal regulation of effector activity by effectors with antagonizing activity has been well documented in *L. pneumophila* infection. For example, the ubiquitin E3 ligase LubX terminates the activity of SidH by targeting it for proteasomal degradation several hours after bacterial uptake^14^. Similarly, the deAMPylase SidD participates in the removal of the small GTPase Rab1 from the LCV 2 h after infection. In each case, temporal regulation is achieved by different expression patterns of the relevant effectors. The LubX protein was not detectable until after several hours postinfection^14^. Similarly, whereas the amount of SidD in infected cells is constant, the amount of the AMPylator SidM decreases as infection proceeds beyond a few hours^35,36^. Interestingly, in the absence of SidD, the association of Rab1 with the LCV only persists for a few extra hours^35,36^, suggesting the presence of host deAMPylases that remove the modification with lower efficiency. In support of this possibility, it has been reported that the mammalian Fic protein FICD possesses deAMPylase activity^37^. Thus, it is possible that one or more host phosphodiesterases capable of reversing SidEs-induced ubiquitination are responsible for the reduction of Ub-Rab33b in cells infected with the Δ*sidJ* mutant. The identification of SidJ forms the foundation for the study of proteins of similar activity, which is of considerable interest if eukaryotic cells also utilize this mechanism of ubiquitination for signaling.

## Materials and Methods

### Media, bacterial strains, plasmid construction and cell lines

*L. pneumophila* strains used in this study were derivatives of the Philadelphia 1 strain Lp02^38^ and were grown and maintained on CYE (charcoal-yeast extract) plates or in ACES-buffered yeast extract (AYE) broth as previously described^38^. The construction of Δ*sidEs* or *sidJ*Δ*sdjA* strains was described previously^17,23^. Genes were cloned into pZL507^39^ for complementation experiments. For infection experiments, *L. pneumophila* was grown to the post-exponential phase (OD_600_ = 3.3-3.8) in AYE broth. For ectopic expression of proteins in mammalian cells, genes were inserted into pEGFPC1 (Clontech) or a 4× Flag vector^20^. All constructs were sequenced to verify the integrity of the inserted genes. HEK293, 293T or COS-1 cells were cultured in Dulbecco's modified minimal Eagle's medium in the presence of 10% FBS. U937 cells were cultured in RPMI 1640 medium supplemented with 10% FBS and differentiated into macrophages as described^40^. *D. discoideum* strains AX4-HDEL-GFP were cultured in HL-5 medium as described earlier^23^. 2 × 10^5^ cells seeded in 24-well plates were infected with an MOI of 5 for immunostaining. Bone marrow-derived macrophages were prepared from 6-week old female A/J mice with L-cell supernatant-conditioned medium as described previously^23^. Macrophages were seeded into 24-well plates 1 day before infection. Cells seeded at 4 × 10^5^ per well were used for intracellular growth experiments. In all cases, 1 h after adding bacteria to cultured cells, infections were synchronized by washing the infected cells three times with warm PBS buffer. Total bacterial counts at indicated time points were determined by plating serially diluted saponin lysates onto bacteriological media.

### Yeast toxicity assays

All yeast strains used in this study were derived from W303^34^; yeast was grown at 30 °C in YPD medium or in appropriate amino acid dropout media in the presence of 2% of glucose or galactose as the sole carbon source. Yeast transformation was performed according to a standard procedure^34^. The yeast strain W303 (pSB157::SdeA), which consistently exhibits a galactose-inducible toxicity has been described earlier^20^. SidJ and its mutants were cloned into p415ADH^41^ and transformed into W303(pSB157::SdeA) for further experiments. To determine the suppressor activity of SidJ, yeast cells cultured in liquid selection medium containing glucose were serially diluted in sterile water (five-fold), and were spotted onto selective medium containing glucose or galactose. Images were acquired after 3-day incubation at 30 °C.

For random mutagenesis of SidJ, DNA of plasmid p415ADH::SidJ was treated with hydroxylamine as previously described^25^. Treated DNA was purified with an affinity column before introducing into the yeast strain W303(pSB157::SdeA). Transformants grown on selective medium containing glucose were tested for the ability to grow on galactose medium. Those failing to grow were screened for the ability to express full-length SidJ by immunoblotting with antibodies specific for SidJ^23^. To eliminate the effects of potential mutations on the plasmid backbone, each mutant was inserted into the vector and the mutations were confirmed by sequencing.

### Transfection, infection, immunoprecipitation, immunostaining and the measurement of secretion by mammalian cells

Lipofectamine 3000 (Thermo Fisher Scientific) was used to transfect cells grown to about 80% confluence. To determine the role of SidJ on the SdeA-mediated modification of Rab33b, GFP-SdeA and 4× Flag-Rab33b were co-transfected with plasmids harboring *sidJ* or its mutants into 293T cells. Cells were collected and lysed with the RIPA buffer^30^ 24 h post transfection. Lysates were subjected to immunoprecipitation with beads coated by the Flag antibody (Sigma-Aldrich) followed by immunoblotting analysis with specific antibodies.

To determine the modification of Rab33b during bacterial infection, HEK293 cells were transfected to express 4× Flag-Rab33b and the FCγRII receptor. 24 h after transfection, cells were infected with opsonized bacteria at an MOI of 10. At the indicated time points, cells were collected and lysed with RIPA buffer^30^. Lysates were subjected to immunoprecipitation with Flag antibody beads as described^20^.

For immunostaining, COS-1 cells were transfected to express Flag-SdeA or Flag-SdeA_E/A_. Cells were fixed 24 h post transfection with paraformaldehyde for 15 min at room temperature. Anti-SdeA antibodies were used at 1:2 000 together with Texas red-conjugated secondary antibody (Invitrogen) to label ectopically expressed SdeA or its SdeA_E/A_ mutant. The ER resident protein calnexin was similarly labeled with a mouse monoclonal antibody and a secondary antibody linked to fluorescein (Invitrogen).

The secretion of SEAP was measured 24 h after cells were transfected with plasmids carrying the relevant genes and the plasmid construct for SEAP expression^42^. Alkaline phosphatase activity was determined with the Tropix phosphalight System kit (Applied Biosystems) per the manufacturer's instructions.

### Protein purification

The *Escheria coli* strain BL21(DE3) was used as the host for expression and purification of recombinant His_6_-4× Flag-Rab33b, His_6_-SdeA and His_6_-sumo-SdeA and His_6_-sumo-SdjA. 30-ml overnight cultures were transferred to 750-ml LB medium supplemented with 100 μg/ml of ampicillin or 30 μg/ml of kanamycin, and was grown to OD_600_ of 0.6∼0.8 prior to the addition of IPTG (isopropyl thio-D-galactopyranoside) to 0.2 mM. The cultures were further incubated at 18 °C in a shaker for 16-18 h. Cells harvested by centrifugation at 12 000× *g* were lysed by sonication in PBS containing protease inhibitors. The bacterial lysates were cleared by centrifugation twice at 12 000× *g* at 4 °C for 15 min. Recombinant proteins were purified with Ni^2+^-NTA beads (Qiagen), and eluted by PBS containing 300 mM imidazole; Purified proteins were dialyzed twice with buffer containing 25 mM Tris-HCl, pH7.5, 150 mM NaCl, 5% glycerol, 1 mM DTT. Protein concentrations were determined by the Bradford assay. GST fusions were similarly purified with glutathione beads. The purity of proteins was greater than 95% as assessed by Coomassie brilliant blue staining. When necessary, the His_6_-sumo tag was removed by ULP1 to produce untagged SdeA^16^.

His_6_-SidJ and its mutant forms were purified from *L. pneumophila* strains harboring pZL507^39^ carrying SidJ or the mutants. Bacteria were grown in 1 l of AYE broth to the post-exponential phase (OD_600_ = 3.3-3.8) in the presence of 0.1 mM IPTG. Cells collected by centrifugation were lysed and protein was purified by the same procedure as described above for *E. coli*.

### *In vitro* de-ubiquitination assays

Ub-Rab33b was produced by incubation of SdeA, His_6_-4Flag-Rab33b and ubiquitin in a buffer containing 50 mM Tris-HCl (pH 7.5), 0.4 mM β-nicotinamide adenine dinucleotide (β-NAD) (Sigma-Aldrich) and 1 mM DTT. The reaction was allowed to proceed at 37 °C for 4 h. Ub-Rab33b was purified by Ni^2+^-NTA beads. Ub-SdeA or Ub-SdeA_178-1 000_ was purified by a similar method from a reaction containing His_6_-SdeA, Flag-ubiquitin and β-NAD. The resulting products were used for SidJ deubiquitination assays as follows: 6 μM of Ub-Rab33b or Ub-SdeA was incubated with 1.6 μM of SidJ or SidJ mutants in 50 mM Tris-HCl (pH 7.5) plus 1 mM DTT. The reaction was allowed to proceed at 37 °C for 2 h or for the indicated times. Reactions were terminated by adding 5×SDS loading buffer.

To test whether SidJ had canonical de-ubiquitination activity, 20 μM suicide inhibitor HA-Ub-VME^19^ was incubated with 0.5 μM of SidJ or 5 μM SdeA_1-193_ in 25 mM Tris-HCl (pH 7.5) at 37 °C for 1 h. Protein samples were resolved by SDS/PAGE and the formation of ubiquitin adducts was visualized by either Coomassie brilliant blue staining or immunoblotting with an HA-specific antibody. The cleavage of canonical polyubiquitinated proteins by SidJ was performed as previously described^19^. In brief, polyubiquitinated and neddylated proteins were produced from 293T cells transfected with pcDNA3-expressing HA-Ubiquitin and Flag-NEDD8, respectively. Cells were lysed and immunoprecipitated with agarose beads coated with α-HA or α-Flag. The beads were resuspended in a cleavage buffer (50 mM Tris-HCl pH 7.5, 100 mM NaCl and 1 mM DTT) and then were divided into aliquots and left untreated or treated with 2 μM of SdeA_1-193_, SidJ or SidJ mutants for 1 h at 37 °C. The beads were then washed twice using the cleavage buffer and boiled for 5 min at 100 °C in 1×SDS-PAGE loading buffer. Proteins were resolved by SDS-PAGE, and polyubiquitinated or neddylated proteins were probed by HA or Flag antibody. For the diubiquitin cleavage assay, 3 μM of SidJ were incubated with 16 μM of diubiquitin in a buffer containing 50 mM Tris-HCl (pH 7.5), 100 mM NaCl, and 1 mM DTT at 37 °C for 2 h. When required, NEM (Sigma) was added at a final concentration of 2 mM. To test the effects of protease inhibitors (ABESF, Bestatin, Pepstatin A and E-64, all from Sigma) on the activity of SidJ, each compound was added at the indicated concentrations to reactions containing 6 μM Ub-Rab33b and 1.6 μM SidJ and the reactions were allowed to proceed for 1 h at 37 °C.

### Saponin extraction and sucrose density gradient ultracentrifugation

To determine protein translocation by *L. pneumophila*, ∼1.0 × 10^7^ of differentiated U937 cells were infected with relevant bacterial strains at an MOI of 5. Infected cells were incubated at 37 °C for 2 h in a 5% CO_2_ incubator. Infected cells were collected and lysed with 0.2% saponin. The lysates were subjected to SDS-PAGE followed by immunoblotting with antibodies against SdeA or SidJ.

To assess the cellular localization of SdeA, 1 × 10^8^ of differentiated U937 cells were infected with relevant *L. pneumophila* strains at an MOI of 5. At the indicated time, the infected cells were suspended in 1 ml of homogenization buffer (20 mM HEPES-KOH, pH 7.2-250 mM sucrose-0.5 mM EGTA) and disrupted in a 7-ml Dounce homogenizer (Wheaton). Unbroken cells and nuclei were removed by centrifugation at 4 °C (3 min, 200*g*). The postnuclear supernatants were layered onto a 20% - 65% sucrose gradient and centrifuged at 100 000*g* for 1.5 h at 4 °C. Fractions were collected and analyzed by immunoblotting after SDS-PAGE.

### Antibodies and immumoblotting

His_6_-SdeC was used to raise rabbit specific antibodies using a standard protocol (Pocono Rabbit Farm & Laboratory). The antibodies were affinity purified as described^17^. For immunoblotting, samples resolved by SDS-PAGE were transferred onto nitrocellulose membranes. After blocking with 5% milk, membranes were incubated with the appropriate primary antibody: anti-GFP (Sigma, cat# G7781), 1:10 000; anti-Flag (Sigma, Cat# F1804), 1: 2 000; anti-PGK (Life Technology, cat# 459250), 1:3 000; anti-SdeA^20^, 1:10 000; anti-Ub (Santa cruz, cat# sc-8017), 1:1 000; anti-His (Sigma, cat# H1029), 1:10 000; anti-tubulin (DSHB, E7), 1:10 000; anti-SidJ, 1:5 000; rabbit anti-calnexin (Abcam, cat# ab22595 for immunoblotting), 1:5 000; mouse anti-calnexin (Thermo Fisher, cat# MA3-027 for immunostaining), 1:200; anti-HA (Santa cruz, cat# sc-7392), 1:1 000; anti-ICDH^39^, 1:20 000. Membranes were then incubated with an appropriate IRDye infrared secondary antibody and scanned using an Odyssey infrared imaging system (Li-Cor's Biosciences). Quantitation of band intensity was performed using Image J.

### Liquid chromatography-tandem mass spectrometry analysis

Protein bands were excised from gels and digested as described previously^43^. The resulting peptides were separated on a C18 reversed phase column connected to a UPLC (ACQUITY, Waters) with a gradient acetonitrile (solvent B) in water (solvent A) both containing 0.1% formic acid: 1% B to 8% B over 5 min and 8% B to 40% B over 95 min at 300 nl/min. The eluted peptides were directly analyzed in a Q-Exactive HF mass spectrometer (Thermo Fisher Scientific), using a 193 nm UVPD (Coherent Existar XS ArF excimer laser) as described previously^44^. The full mass spectra were collected in a range of 350-1 800 m/z with a 60 k resolution and the top 15 most intense ions were submitted for UVPD using a single 2 mJ pulse per MS/MS spectrum, before being dynamically excluded for 10 s.

## Author Contributions

JQ and ZQL designed the experiments; JQ, YL, XF and KP performed the experiments; KY, XL, PDP, JBS and ESN performed the mass spectrometric analyses; CD provided resources; JQ and ZQL analyzed the data and wrote the paper, and all authors contributed editorial input. ZQL supervised the project.

## Competing Financial Interests

The authors declare no competing financial interests.

## Supplementary Information

Supplementary information, Figure S1 The property of SidJ homolog SdjA.

Supplementary information, Figure S2 Deletion analysis of SidJ and the interactions between SidJ and ubiquitin.

Supplementary information, Figure S3 The cleavage of differently linked diubiquitins by SidJ and its mutants.

Supplementary information, Figure S4 The activity of SidJ cysteine mutants.

Supplementary information, Figure S5 The activity of SidJ in deconjugating neddylated proteins, its sensitivity to protease inhibitors and substrate preference.

Supplementary information, Figure S6 Mass spectrometric analysis of unmodified ubiquitin peptide- E_34_GIPPDQQR_42_- in ubiquitin from different sources.

Supplementary information, Figure S7 Cellular localization of SidJ.

Supplementary information, Figure S8 Detection of endogenous proteins by antibodies specific for SdeA and SdeC.

## References

[bib1] Hershko A, Ciechanover A, Varshavsky A. Basic Medical Research Award. The ubiquitin system. Nat Med 2000; 6:1073–1081. 11017125 10.1038/80384

[bib2] Popovic D, Vucic D, Dikic I. Ubiquitination in disease pathogenesis and treatment. Nat Med 2014; 20:1242–1253. 25375928 10.1038/nm.3739

[bib3] Huibregtse JM, Scheffner M, Beaudenon S, Howley PM. A family of proteins structurally and functionally related to the E6-AP ubiquitin-protein ligase. Proc Natl Acad Sci USA 1995; 92:2563–2567. 7708685 10.1073/pnas.92.7.2563 PMC42258

[bib4] Maculins T, Fiskin E, Bhogaraju S, Dikic I. Bacteria-host relationship: ubiquitin ligases as weapons of invasion. Cell Res 2016; 26:499–510. 26964724 10.1038/cr.2016.30 PMC4822128

[bib5] Newton HJ, Ang DK, van Driel IR, Hartland EL. Molecular pathogenesis of infections caused by *Legionella pneumophila*. Clin Microbiol Rev 2010; 23:274–298. 20375353 10.1128/CMR.00052-09 PMC2863363

[bib6] Xu L, Luo ZQ. Cell biology of infection by *Legionella pneumophila*. Microbes Infect 2013; 15:157–167. 23159466 10.1016/j.micinf.2012.11.001 PMC3558624

[bib7] Hubber A, Kubori T, Nagai H. Modulation of the ubiquitination machinery by Legionella. Curr Top Microbiol Immunol 2013; 376:227–247. 23918174 10.1007/82_2013_343

[bib8] Dorer MS, Kirton D, Bader JS, Isberg RR. RNA interference analysis of Legionella in *Drosophila* cells: exploitation of early secretory apparatus dynamics. PLoS Pathog 2006; 2:e34. 16652170 10.1371/journal.ppat.0020034 PMC1447669

[bib9] Huang L, Boyd D, Amyot WM, et al. The E block motif is associated with *Legionella pneumophila* translocated substrates. Cellular Microbiol 2010; 13:227–245. 10.1111/j.1462-5822.2010.01531.x PMC3096851 20880356

[bib10] Lifshitz Z, Burstein D, Peeri M, et al. Computational modeling and experimental validation of the Legionella and Coxiella virulence-related type-IVB secretion signal. Proc Natl Acad Sci USA 2013; 110:E707–E715. 23382224 10.1073/pnas.1215278110 PMC3581968

[bib11] Zhu W, Banga S, Tan Y, et al. Comprehensive identification of protein substrates of the Dot/Icm type IV transporter of *Legionella pneumophila*. PLoS One 2011; 6:e17638. 21408005 10.1371/journal.pone.0017638 PMC3052360

[bib12] Habyarimana F, Al-Khodor S, Kalia A, et al. Role for the Ankyrin eukaryotic-like genes of *Legionella pneumophila* in parasitism of protozoan hosts and human macrophages. Environ Microbiol 2008; 10:1460–1474. 18279343 10.1111/j.1462-2920.2007.01560.x

[bib13] Ensminger AW, Isberg RR. E3 ubiquitin ligase activity and targeting of BAT3 by multiple *Legionella pneumophila* translocated substrates. Infect Immun 2010; 78:3905–3919. 20547746 10.1128/IAI.00344-10 PMC2937443

[bib14] Kubori T, Shinzawa N, Kanuka H, Nagai H. Legionella metaeffector exploits host proteasome to temporally regulate cognate effector. PLoS Pathog 2010; 6:e1001216. 21151961 10.1371/journal.ppat.1001216 PMC2996335

[bib15] Lin YH, Doms AG, Cheng E, Kim B, Evans TR, Machner MP. Host cell-catalyzed S-palmitoylation mediates golgi targeting of the legionella ubiquitin ligase GobX. J Biol Chem 2015; 290:25766–25781. 26316537 10.1074/jbc.M115.637397 PMC4646218

[bib16] Hsu F, Luo X, Qiu J, et al. The Legionella effector SidC defines a unique family of ubiquitin ligases important for bacterial phagosomal remodeling. Proc Natl Acad Sci USA 2014; 111:10538–10543. 25006264 10.1073/pnas.1402605111 PMC4115505

[bib17] Luo ZQ, Isberg RR. Multiple substrates of the *Legionella pneumophila* Dot/Icm system identified by interbacterial protein transfer. Proc Natl Acad Sci USA 2004; 101:841–846. 14715899 10.1073/pnas.0304916101 PMC321768

[bib18] Bardill JP, Miller JL, Vogel JP. IcmS-dependent translocation of SdeA into macrophages by the *Legionella pneumophila* type IV secretion system. Mol Microbiol 2005; 56:90–103. 15773981 10.1111/j.1365-2958.2005.04539.x

[bib19] Sheedlo MJ, Qiu J, Tan Y, Paul LN, Luo ZQ, Das C. Structural basis of substrate recognition by a bacterial deubiquitinase important for dynamics of phagosome ubiquitination. Proc Natl Acad Sci USA 2015; 112:15090–15095. 26598703 10.1073/pnas.1514568112 PMC4679006

[bib20] Qiu J, Sheedlo MJ, Yu K, et al. Ubiquitination independent of E1 and E2 enzymes by bacterial effectors. Nature 2016; 533:120–124. 27049943 10.1038/nature17657 PMC4905768

[bib21] Bhogaraju S, Kalayil S, Liu Y, et al. Phosphoribosylation of ubiquitin promotes serine ubiquitination and impairs conventional ubiquitination. Cell 2016; 167:1636–1649 e1613. 27912065 10.1016/j.cell.2016.11.019

[bib22] Kotewicz KM, Ramabhadran V, Sjoblom N, et al. A single legionella effector catalyzes a multistep ubiquitination pathway to rearrange tubular endoplasmic reticulum for replication. Cell Host Microbe 2017; 21:169–181. 28041930 10.1016/j.chom.2016.12.007 PMC5300936

[bib23] Liu Y, Luo ZQ. The *Legionella pneumophila* effector SidJ is required for efficient recruitment of endoplasmic reticulum proteins to the bacterial phagosome. Infect Immun 2007; 75:592–603. 17101649 10.1128/IAI.01278-06 PMC1828518

[bib24] Jeong KC, Sexton JA, Vogel JP. Spatiotemporal regulation of a *Legionella pneumophila* T4SS substrate by the metaeffector SidJ. PLoS Pathog 2015; 11:e1004695. 25774515 10.1371/journal.ppat.1004695 PMC4361747

[bib25] Luo ZQ, Farrand SK. Signal-dependent DNA binding and functional domains of the quorum-sensing activator TraR as identified by repressor activity. Proc Natl Acad Sci USA 1999; 96:9009–9014. 10430886 10.1073/pnas.96.16.9009 PMC17723

[bib26] Reyes-Turcu FE, Ventii KH, Wilkinson KD. Regulation and cellular roles of ubiquitin-specific deubiquitinating enzymes. Annu Rev Biochem 2009; 78:363–397. 19489724 10.1146/annurev.biochem.78.082307.091526 PMC2734102

[bib27] Pink JJ, Wuerzberger-Davis S, Tagliarino C, et al. Activation of a cysteine protease in MCF-7 and T47D breast cancer cells during beta-lapachone-mediated apoptosis. Exp Cell Res 2000; 255:144–155. 10694431 10.1006/excr.1999.4790

[bib28] Borodovsky A, Kessler BM, Casagrande R, Overkleeft HS, Wilkinson KD, Ploegh HL. A novel active site-directed probe specific for deubiquitylating enzymes reveals proteasome association of USP14. EMBO J 2001; 20:5187–5196. 11566882 10.1093/emboj/20.18.5187 PMC125629

[bib29] Mayfield JE, Robinson MR, Cotham VC, et al. Mapping the phosphorylation pattern of drosophila melanogaster RNA polymerase ii carboxyl-terminal domain using ultraviolet photodissociation mass spectrometry. ACS Chem Biol 2017; 12:153–162. 28103682 10.1021/acschembio.6b00729 PMC5376213

[bib30] Banga S, Gao P, Shen X, et al. *Legionella pneumophila* inhibits macrophage apoptosis by targeting pro-death members of the Bcl2 protein family. Proc Natl Acad Sci USA 2007; 104:5121–5126. 17360363 10.1073/pnas.0611030104 PMC1829273

[bib31] Hsu F, Zhu W, Brennan L, Tao L, Luo ZQ, Mao Y. Structural basis for substrate recognition by a unique Legionella phosphoinositide phosphatase. Proc Natl Acad Sci USA 2012; 109:13567–13572. 22872863 10.1073/pnas.1207903109 PMC3427105

[bib32] Losick VP, Isberg RR. NF-kappaB translocation prevents host cell death after low-dose challenge by *Legionella pneumophila*. J Exp Med 2006; 203:2177–2189. 16940169 10.1084/jem.20060766 PMC2118400

[bib33] Abu-Zant A, Jones S, Asare R, et al. Anti-apoptotic signalling by the Dot/Icm secretion system of *L. pneumophila*. Cell Microbiol 2007; 9:246–264. 16911566 10.1111/j.1462-5822.2006.00785.x

[bib34] Tan Y, Arnold RJ, Luo ZQ. *Legionella pneumophila* regulates the small GTPase Rab1 activity by reversible phosphorylcholination. Proc Natl Acad Sci USA 2011; 108:21212–21217. 22158903 10.1073/pnas.1114023109 PMC3248503

[bib35] Neunuebel MR, Chen Y, Gaspar AH, Backlund PS. Jr, Yergey A, Machner MP. De-AMPylation of the small GTPase Rab1 by the pathogen *Legionella pneumophila*. Science 2011; 333:453–456. 21680813 10.1126/science.1207193 PMC3209958

[bib36] Tan Y, Luo ZQ. *Legionella pneumophila* SidD is a deAMPylase that modifies Rab1. Nature 2011; 475:506–509. 21734656 10.1038/nature10307 PMC3146296

[bib37] Preissler S, Rato C, Perera LA, Saudek V, Ron D. FICD acts bifunctionally to AMPylate and de-AMPylate the endoplasmic reticulum chaperone BiP. Nat Struct Mol Biol 2017; 24:23–29. 27918543 10.1038/nsmb.3337 PMC5221731

[bib38] Berger KH, Isberg RR. Two distinct defects in intracellular growth complemented by a single genetic locus in *Legionella pneumophila*. Mol Microbiol 1993; 7:7–19. 8382332 10.1111/j.1365-2958.1993.tb01092.x

[bib39] Xu L, Shen X, Bryan A, Banga S, Swanson MS, Luo ZQ. Inhibition of host vacuolar H^+^-ATPase activity by a *Legionella pneumophila* effector. PLoS Pathog 2010; 6:e1000822. 20333253 10.1371/journal.ppat.1000822 PMC2841630

[bib40] Conover GM, Derre I, Vogel JP, Isberg RR. The *Legionella pneumophila* LidA protein: a translocated substrate of the Dot/Icm system associated with maintenance of bacterial integrity. Mol Microbiol 2003; 48:305–321. 12675793 10.1046/j.1365-2958.2003.03400.x

[bib41] Mumberg D, Muller R, Funk M. Yeast vectors for the controlled expression of heterologous proteins in different genetic backgrounds. Gene 1995; 156:119–122. 7737504 10.1016/0378-1119(95)00037-7

[bib42] Pan X, Luhrmann A, Satoh A, Laskowski-Arce MA, Roy CR. Ankyrin repeat proteins comprise a diverse family of bacterial type IV effectors. Science 2008; 320:1651–1654. 18566289 10.1126/science.1158160 PMC2514061

[bib43] Shevchenko A, Tomas H, Havlis J, Olsen JV, Mann M. In-gel digestion for mass spectrometric characterization of proteins and proteomes. Nat Protoc 2006; 1:2856–2860. 17406544 10.1038/nprot.2006.468

[bib44] Fort KL, Dyachenko A, Potel CM, et al. Implementation of ultraviolet photodissociation on a benchtop Q exactive mass spectrometer and its application to phosphoproteomics. Anal Chem 2016; 88:2303–2310. 26760441 10.1021/acs.analchem.5b04162

