# Supplementary material for: A unique deubiquitinase that deconjugates phosphoribosyl-linked protein ubiquitination
Source: Cell Res. 2017 May 12;27(7):865–81. doi: 10.1038/cr.2017.66 (PMC5518988; doi:10.1038/cr.2017.66)
Supplement: Supplementary information, Figure S1 — The property of SidJ homolog SdjA. [file cr201766x1.pdf]

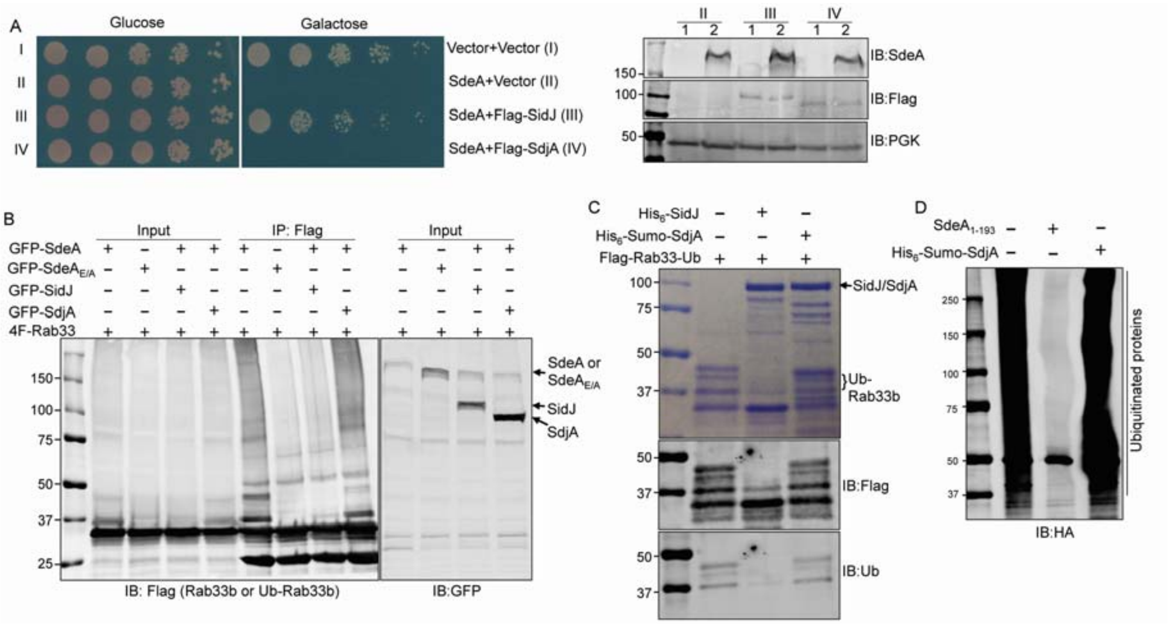

Figure S1 The property of SidJ homolog SdjA. A. SdjA did not suppress the yeast toxicity induced by SdeA. The yeast strain expressing SdeA from the galactose-inducible promoter was transformed with plasmids that direct the expression of Flag-tagged SidJ or SdjA. Yeast cells diluted in water were spotted onto medium containing glucose or galactose and the growth of the yeast cells were assessed 3 days post spotting (left panel). The expression of SdeA, SidJ and SdjA was evaluated by immunoblotting of lysates of the yeast strains grown in glucose (1) or galactose (2) medium with antibodies specific for Flag or SdeA (right panel). The yeast metabolic protein 3-phosphoglycerate kinase (PGK) was probed as a loading control (right panel). **B.** SdjA did not counteract Rab33b ubiquitination induced by SdeA. Combinations of plasmids were transfected to express Flag-Rab33b, SidJ or SdjA in HEK293 cells. Immunoprecipitates obtained with the Flag antibody-coated beads were probed with the Flag antibody. Note that co-expression of SdjA did not cause reduction of Ub-Flag-Rab33b. **C.** SdjA cannot remove ubiquitin from Ub-Rab33b. 1.6 μM of recombinant SidJ or SdjA was added to reactions containing 6 μM of Ub-Flag-Rab33b. After

incubation for 2 h, terminated reactions resolved by SDS-PAGE were probed by Coomassie staining (top panel) or by immunoblotting with antibody specific for the Flag (middle panel) or ubiquitin (lower panel). Note that treatment with SidJ but not SdjA led to reduction in ubiquitinated Rab33b. **D**. SdjA cannot remove ubiquitin from proteins modified by conventional ubiquitination. Ubiquitinated proteins purified from 293T cells transfected to express HA-ubiquitin were incubated with $SdeA_{1-193}$ or His_6-sumo-SdjA for 2 h and the ubiquitination status of the samples was probed by immunoblotting with an antibody specific for the HA tag. In each panel, similar results were obtained in at least three independent experiments.
