# Supplementary material for: A unique deubiquitinase that deconjugates phosphoribosyl-linked protein ubiquitination
Source: Cell Res. 2017 May 12;27(7):865–81. doi: 10.1038/cr.2017.66 (PMC5518988; doi:10.1038/cr.2017.66)
Supplement: Supplementary information, Figure S2 — Deletion analysis of SidJ and the interactions between SidJ and ubiquitin. [file cr201766x2.pdf]

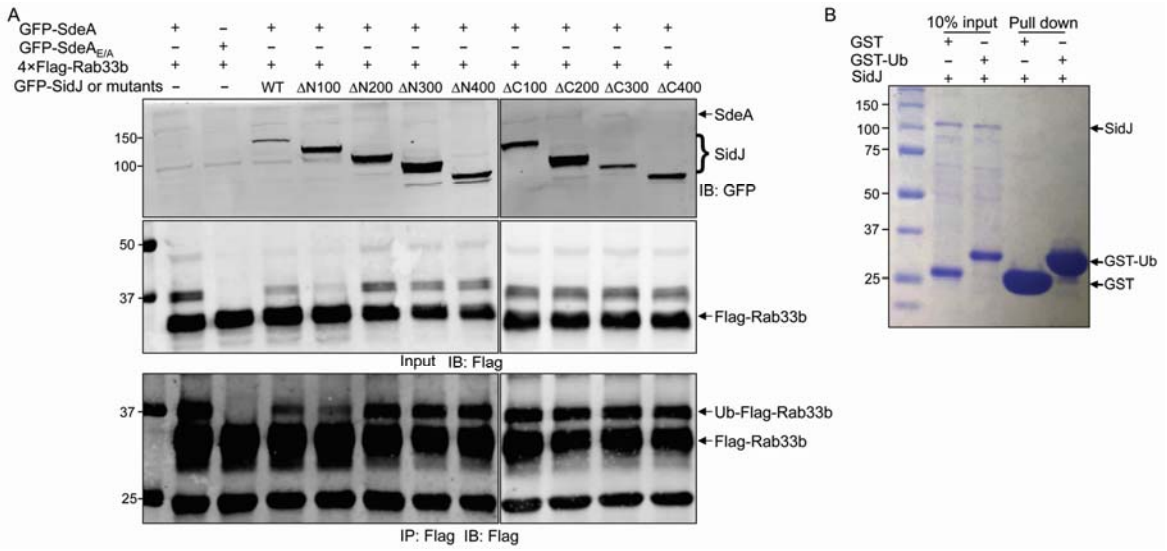

Figure S2 Deletion analysis of SidJ and the interactions between SidJ and ubiquitin. A. GFP fusion of SidJ and its deletion mutants were co-expressed with SdeA and Rab33b for 24 h. Lysates of transfected cells were probed for the expression of SidJ and its derivatives (upper panel) and for ubiquitination of Rab33b in total cell lysates (middle panel) or after immunoprecipitation with beads coated with the Flag antibody (lower panel). Note that only the wild type and the SidJΔN100 mutants caused reduction in Ub-Rab33b. A sample transfected with the $SdeA_{E/A}$ mutant unable to activate ubiquitin was included as a control (2nd lane). **B**. SidJ does not detectably interact with ubiquitin. GST-ubiquitin or GST was incubated with SidJ and GST beads were used to capture the potential complex formed between SidJ and ubiquitin. Samples were resolved by SDS-PAGE prior to being stained with Coomassie brilliant blue. Note that GST-ubiquitin did not detectably retain SidJ. In each panel, similar results were obtained in three independent experiments.
