# Supplementary material for: A unique deubiquitinase that deconjugates phosphoribosyl-linked protein ubiquitination
Source: Cell Res. 2017 May 12;27(7):865–81. doi: 10.1038/cr.2017.66 (PMC5518988; doi:10.1038/cr.2017.66)
Supplement: Supplementary information, Figure S3 — The cleavage of differently linked diubiquitins by SidJ and its mutants. [file cr201766x3.pdf]

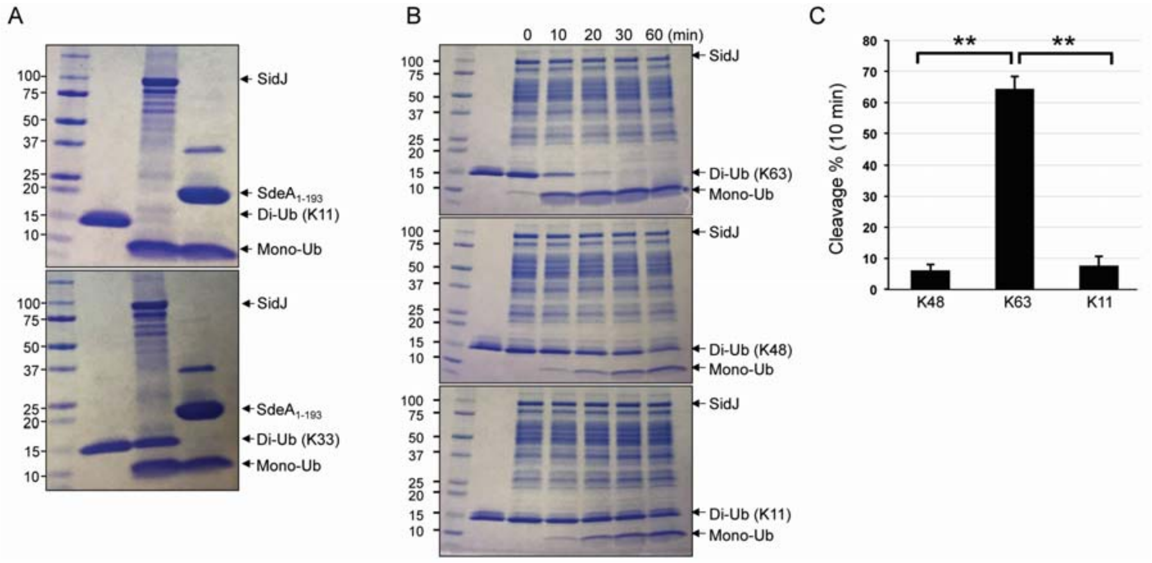

Figure S3 The cleavage of differently linked diubiquitins by SidJ and its mutants.

A. 16 µM of K11- (top panel) or K-33 linked (bottom panel) diubiquitin was incubated with 3 µM of SidJ for 2 h at 37°C. The cleavage was evaluated by Coomassie staining. In each case, the diubiquitin was completely cleaved by $SdeA_{1-193}$. Note that a considerable amount K33-linked diubiquitin was not digested. Similar results were obtained in two independent experiments. **B**. Time-dependent cleavage of diubiquitins by SidJ. 16 µM of K63-, K48- or K11-linked diubiquitins was incubated with 3 µM of SidJ for the indicated times. Proteins resolved by SDS-PAGE were detected by Coomassie staining. **C**. Quantitation of diubiquitin cleavage by SidJ after 10 min reaction. The intensity of diubiquitin bands from three independent experiments was measured to calculate the percentage of cleavage. Error bars, standard error (s.e.). **, $p<0.01$.
