# Supplementary material for: A unique deubiquitinase that deconjugates phosphoribosyl-linked protein ubiquitination
Source: Cell Res. 2017 May 12;27(7):865–81. doi: 10.1038/cr.2017.66 (PMC5518988; doi:10.1038/cr.2017.66)
Supplement: Supplementary information, Figure S4 — The activity of SidJ cysteine mutants. [file cr201766x4.pdf]

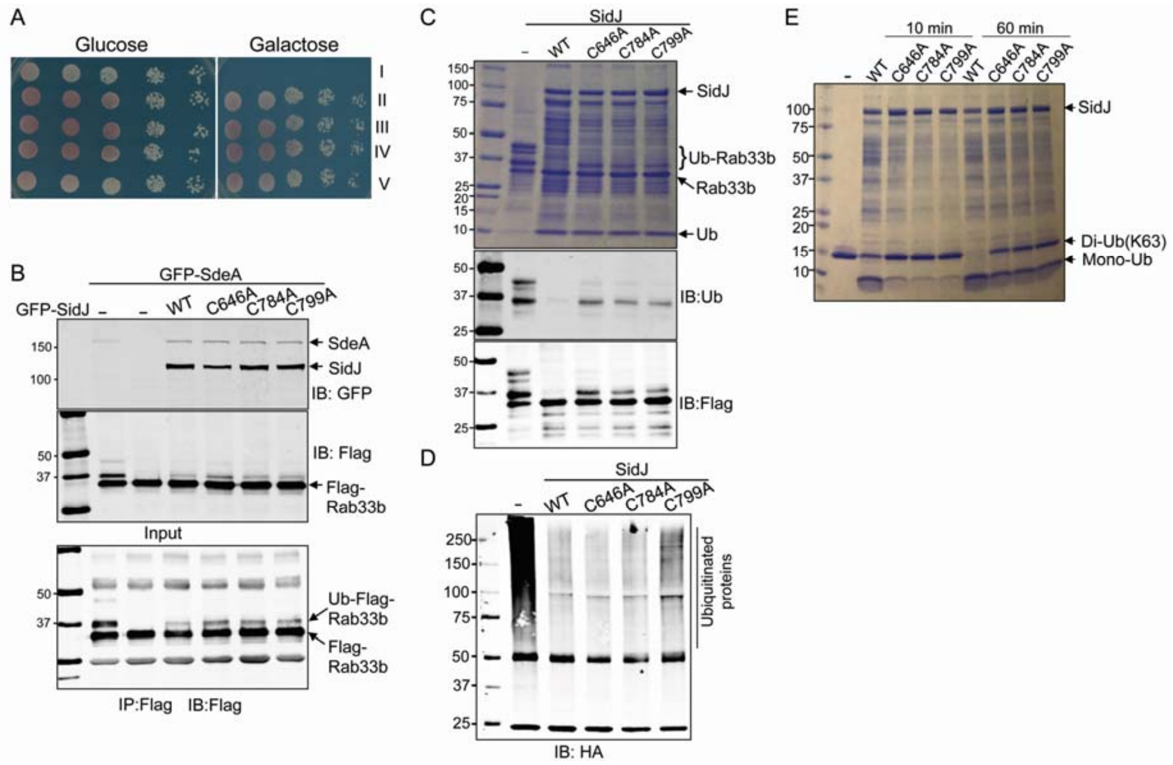

Figure S4 The activity of SidJ cysteine mutants. A. The suppression of SdeA yeast toxicity by SidJ cysteine mutants. Plasmids carrying SidJ or the cysteine mutants were transformed into the yeast strain that harbors galactose-inducible SdeA. Suppressor activity was examined by spotting diluted yeast cells onto minimal medium containing glucose (left panel) or galactose (right panel). Images were acquired after incubation at 30°C for 3 d. Yeast strains: I, SdeA+Vector; II, SdeA+SidJ; III, SdeA+SidJ$_{C646A}$; IV, SdeA+SidJ$_{C784A}$; V, SdeA+SidJ$_{C799A}$. **B**. The cysteine mutants of SidJ still counteract the activity of SdeA in mammalian cells. GFP fusions of SdeA, SidJ and its cysteine mutants were coexpressed with Flag-Rab33b in 293T cells. Cell lysates were probed for GFP-SdeA, GFP-SidJ and its mutants (upper panel) and for ubiquitinated Rab33b indicated by molecular weight shift. Ubiquitinated Rab33b was also probed after enrichment by immunoprecipitation with beads coated with the Flag antibody (lower panel). **C.** *In vitro* activity of SidJ cysteine mutants in deubiquitinating Ub-Rab33b. 1.6

μM of recombinant SidJ or its cysteine mutants were incubated with 6 μM of Ub-Rab33b for 2 h at 37ºC and the removal of ubiquitin from the substrate was determined by Coomassie staining (upper panel) or by immunoblotting with antibodies specific for ubiquitin (middle panel) and Flag (lower panel). Note that in samples receiving the mutants, ubiquitinated Rab33b was still readily detectable. **D.** The removal of ubiquitin attached by the canonical mechanism by SidJ cysteine mutants. 2 μM of recombinant SidJ or its cysteine mutants were incubated with ubiquitinated proteins isolated from 293T cells transfected to express HA-ubiquitin and the ubiquitin signals were detected by immunoblotting with an HA-specific antibody. **E**. The cleavage of K63-linked diubiquitin by cysteine mutants of SidJ. 16 μM of K63-linked diubiquitin was incubated with 3 μM of SidJ or its cysteine mutants for 10 or 60 min. The cleavage was detected by Coomassie staining. Similar results were obtained in two independent experiments.
