# Supplementary material for: A unique deubiquitinase that deconjugates phosphoribosyl-linked protein ubiquitination
Source: Cell Res. 2017 May 12;27(7):865–81. doi: 10.1038/cr.2017.66 (PMC5518988; doi:10.1038/cr.2017.66)
Supplement: Supplementary information, Figure S5 — The activity of SidJ in deconjugating neddylated proteins, its sensitivity to protease inhibitors and substrate preference. [file cr201766x5.pdf]

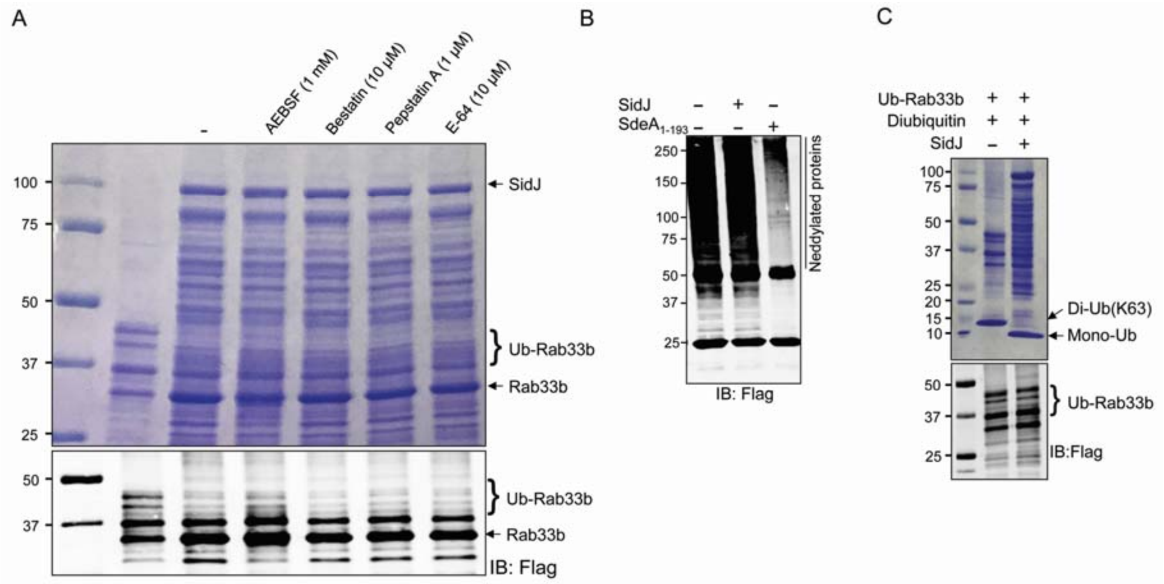


**Figure S5 The activity of SidJ in deconjugating neddylated proteins, its sensitivity to protease inhibitors and substrate preference. A.** SidJ is not sensitive to four protease inhibitors. Protease inhibitors at the indicated concentrations were added to reactions containing 1.6 μM SidJ and 6 μM Ub-Rab33b. The reactions were allowed to proceed for 1 h at 37ºC. Samples resolved by SDS-PAGE were detected by immunoblotting with a Flag-specific antibody. **B**. SidJ cannot remove NEDD8 from modified proteins. Neddylated proteins isolated from 293T cells transfected to express Flag-NEDD8 were incubated with 2 μM of SidJ or $SdeA_{Dub}$ and the NEDD8 signals were detected by immunoblotting with a Flag antibody. **C**. SidJ cleaves K63-linked diubiquitin faster than deconjugating ubiquitin from Ub-Rab33b in reactions containing both substrates. 3 μM of SidJ was incubated with equal molar (9 μM) of Ub-Rab33b and K63-linked ubiquitin for 15 min. Deubiquitination and cleavage of diubiquitin were probed by Coomassie staining (top panel) or by immunoblotting with antibody specific for Flag (Flag-Ub was used to prepare Ub-Rab33b) (lower panel). Note that in this

reaction duration all of the diubiquitin had been hydrolyzed, yet most Ub-Rab33b remained intact.
