# Supplementary material for: A unique deubiquitinase that deconjugates phosphoribosyl-linked protein ubiquitination
Source: Cell Res. 2017 May 12;27(7):865–81. doi: 10.1038/cr.2017.66 (PMC5518988; doi:10.1038/cr.2017.66)
Supplement: Supplementary information, Figure S6 — Mass spectrometric analysis of unmodified ubiquitin peptide- E34GIPPDQQR42- in ubiquitin from different sources. [file cr201766x6.pdf]

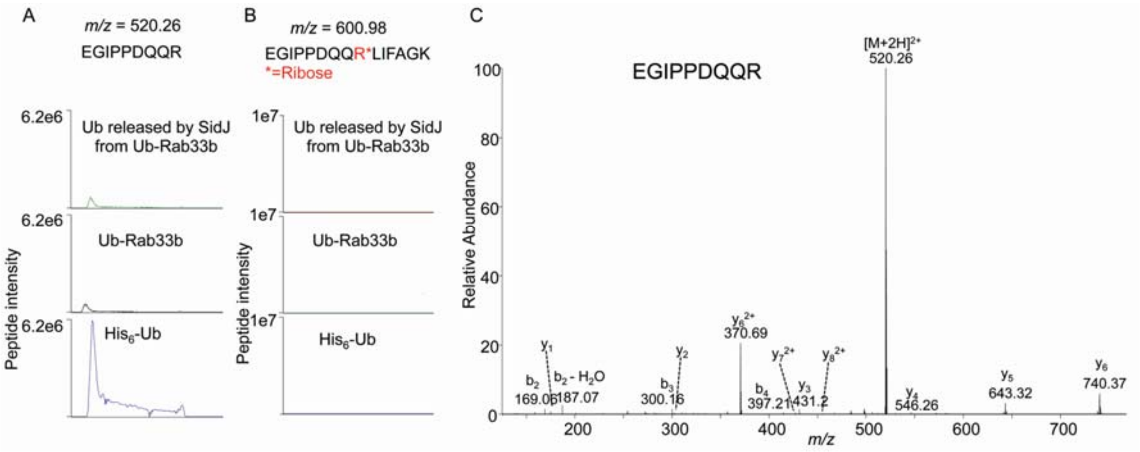

Figure S6 Mass spectrometric analysis of unmodified ubiquitin peptide-E$_{34}$GIPPDQQR$_{42}$- in ubiquitin from different sources. A. Extracted ion chromatograms at m/z 520.26 corresponding to the doubly-charged peptide from unmodified ubiquitin -E$_{34}$GIPPDQQR$_{42}$-. His$_6$-Ub was included as a control, and Rab33b-Ub was used to determine the amount of unreacted ubiquitin in the sample. **B.** Extracted ion chromatograms at m/z 600.98 corresponding to the triply-charged peptide from ubiquitin -E$_{34}$GIPPDQQRLIFAGK$_{48}$- containing a ribosyl moiety. Note that no such modification was detected. **C.** Ultraviolet photon dissociation (UVPD) mass spectrum of the ubiquitin peptide -E$_{34}$GIPPDQQR$_{42}$-.
