# Supplementary material for: A unique deubiquitinase that deconjugates phosphoribosyl-linked protein ubiquitination
Source: Cell Res. 2017 May 12;27(7):865–81. doi: 10.1038/cr.2017.66 (PMC5518988; doi:10.1038/cr.2017.66)
Supplement: Supplementary information, Figure S7 — Cellular localization of SidJ. [file cr201766x7.pdf]

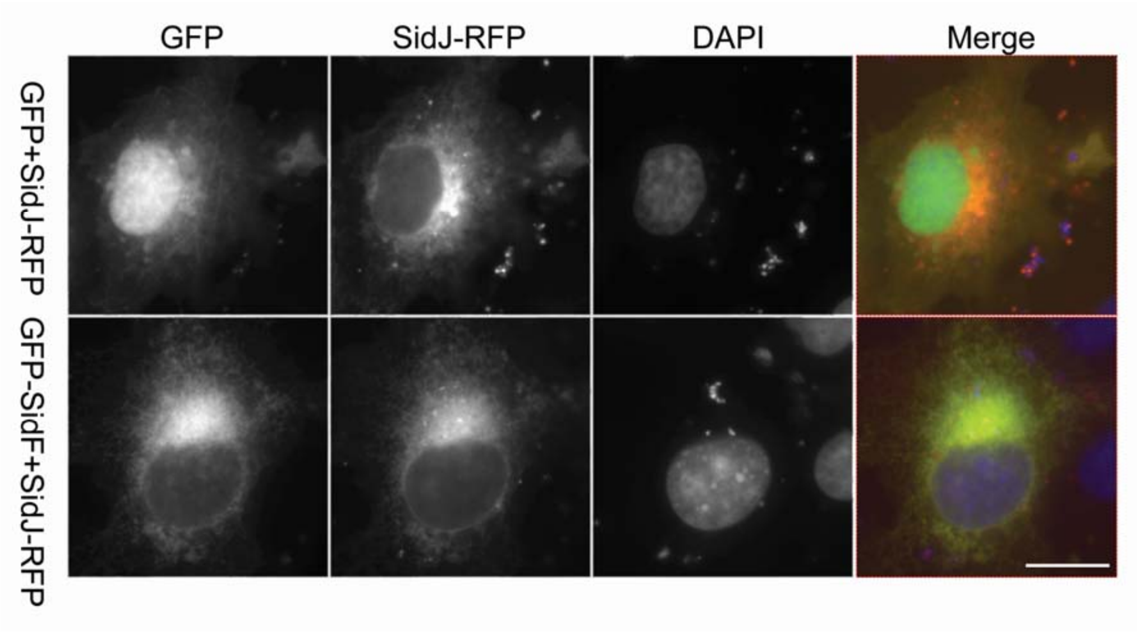

Figure S7 Cellular localization of SidJ. COS-1 cells were transfected to express RFP-SidJ with GFP or GFP-SidF, an effector that localizes to the ER. Representative images were acquired by an IX-81 Olympus fluorescence microscope. Similar patterns of distribution were seen in two independent experiments. Bar, 5 μm.
