# Supplementary material for: A unique deubiquitinase that deconjugates phosphoribosyl-linked protein ubiquitination
Source: Cell Res. 2017 May 12;27(7):865–81. doi: 10.1038/cr.2017.66 (PMC5518988; doi:10.1038/cr.2017.66)
Supplement: Supplementary information, Figure S8 — Detection of endogenous proteins by antibodies specific for SdeA and SdeC. [file cr201766x8.pdf]

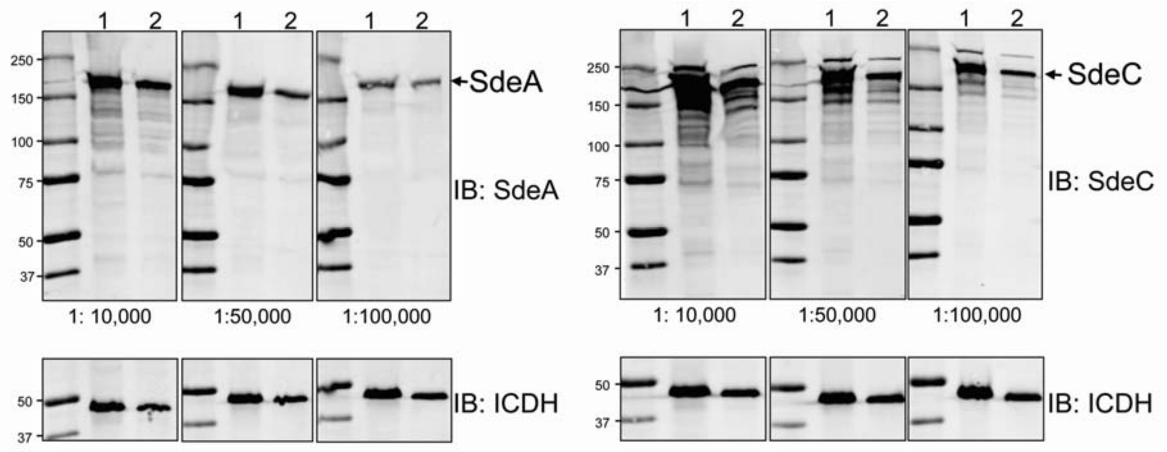

Figure S8 Detection of endogenous proteins by antibodies specific for SdeA and SdeC. Cells of wild type *L. pneumophila* grown to OD_{600} of 3.4 were lysed and the SDS-PAGE loading buffer soluble fraction was resolved prior to immunoblotting with antibodies against SdeA (left panel) or SdeC (right panel). The ratio below each blot indicates the dilution used for the detection. In each case, the sample was loaded with 0.05 OD (approximately $5x10^7$) (1) or 0.025 OD (approximately $2.5x10^7$) bacteria (2), respectively. The metabolic enzyme isocitrate dehydrogenase (ICDH) was probed as loading controls.
